# How does intrauterine crowding affect locomotor performance in newborn pigs? A study of force generating capacity and muscle composition of the hind limb

**DOI:** 10.1371/journal.pone.0209233

**Published:** 2018-12-14

**Authors:** Charlotte Vanden Hole, Silke Cleuren, Chris Van Ginneken, Sara Prims, Miriam Ayuso, Steven Van Cruchten, Peter Aerts

**Affiliations:** 1 Laboratory of Applied Veterinary Morphology, Department of Veterinary Sciences, Faculty of Biomedical, Pharmaceutical and Veterinary Sciences, University of Antwerp, Wilrijk, Belgium; 2 Laboratory of Functional Morphology, Department of Biology, Faculty of Sciences, University of Antwerp, Wilrijk, Belgium; 3 Department of Movement and Sports Sciences, Faculty of Medicine and Health Sciences, Ghent University, Ghent, Belgium; INIA, SPAIN

## Abstract

Intrauterine crowding (IUC) considerably influences postnatal traits in a polytocous species such as the pig. Previously, our group described how IUC affects locomotion during the piglet’s first days of life (until 96 h after birth). We noted a reduced motor performance in piglets with a low birth weight and low vitality (L piglets), compared to piglets with a normal birth weight and normal vitality (N piglets), indicating L piglets are unable to produce enough force. Our current study investigates whether this observed force deficit in L piglets is caused by a reduced force generating capacity in the muscles and/or a lower percentage of type II (fast-contracting) fibers. Volume and fiber length of the main extensor muscles of the hind limb were used to estimate the physiological cross-sectional area (PSCA) and hence calculate the maximal isometric force generating capacity (F_iso-max_) of the hind limb. To check for developmental differences between the muscles of L and N piglets, F_iso-max_ was normalized to body weight (BW), thus yielding a dimensionless variable F’_iso-max_. To check for differences in muscle composition, m. vastus lateralis was stained immunohistochemically in order to determine the percentage of type II fibers through image analysis. Our results indicate that L piglets have a reduced absolute force generating capacity due to a lesser muscle mass, compared to N piglets. However, when normalized to BW L piglets actually show a larger force generating capacity, suggesting their muscles are more voluminous, given their body mass, than those of N piglets. However, no differences between L and N piglets were detected with regard to muscle composition of the m. vastus lateralis. Based on our data, we can say that neither normalized force generating capacity, nor muscle composition (of the m. vastus lateralis) can explain the observed force deficit in L piglets and as such the effect of IUC on locomotor performance.

## Introduction

Polytocous species, such as the domestic pig (*Sus scrofa domesticus*), conceive large numbers of offspring. However, limited uterine space and placental area (and as such blood flow to the fetus) causes intra-uterine competition amongst the piglets. This (at least partially) explains why increased crowding in the uterus leads to (more) small piglets at birth [1, 2]. Although the wild boar (*Sus scrofa* [3]) is also a polycotous species, intrauterine crowding (IUC) and its effect are exacerbated in modern breeding sows that are genetically selected for producing extremely large litters (>14) [4]. As a consequence, heterogeneity in the offspring’s birth weight and an increased number of small piglets are observed [5].

Research already showed that IUC greatly influences postnatal traits in pigs [6–12]. Recently, our group described how IUC affects locomotion during the piglet’s first days of life [13]. In this study, spatio-temporal gait variables were compared between piglets with a normal birth weight and normal vitality (N piglets) and piglets with a low birth weight and low vitality (L piglets). Among other findings, we observed a reduced motor performance (measured by speed and its components stride length and stride frequency) in L piglets. To increase its performance, an L piglet would have to move its limbs more rapidly and reduce the time that its feet are in contact with the ground. However, to do this, the piglet’s muscles need to generate greater forces and contract more rapidly [14]. The observed inability to increase performance in L piglets suggests they are unable to produce enough force (thus showing a force deficit) and/or increase muscle contraction velocity.

To unravel the underlying mechanisms through which IUC and its associated birth weight variability affect locomotion, the development of the musculoskeletal system and its control system as well as the energy available for locomotion, should be carefully studied. Some reports on the m. semitendinosus, already show that IUC results in smaller muscle cross-sectional areas and a lower number of myofibers [9, 12]. Nevertheless, the description of the musculoskeletal system in view of IUC and motor performance is far from complete.

This study aims at helping to close the abovementioned gap by investigating whether the force deficit in L piglets is caused by a lower force generating capacity. To this end, muscle volume and fiber length are combined to estimate the physiological cross-sectional area (PCSA) and hence the maximal isometric force generation capacity. Given their lower body mass (BM), it would make sense that L piglets are more slender (i.e. less muscular) than N piglets. However, they might also be overall smaller (i.e. have shorter legs) than N piglets. Therefore, hind limb length is considered in addition to BM and body mass index (BMI). We propose that a developmental delay of the motor performance of L piglets is linked to a lower relative (to size) force generating capacity for L piglets when compared to N piglets.

However, it is important to keep in mind that muscle architecture and force transmission are complex and cannot be described using gross dissection alone [15]. The observed force deficit in L piglets might as well be caused by a different fiber composition of the muscle, compared to N piglets. To that end we investigate the composition of the m. quadriceps femoris (m. vastus lateralis) by immunohistochemical fiber typing. Studies on humans [16–18] and rats [19–21] have clearly described a relation between maximum force produced by a fiber and its type, with type II fibers being able to produce more force than type I. As both main types of muscle fibers (type I and II; for a review on fiber types see [22]) have their specific properties (i.e. slow-twitch oxidative and fast-twitch, respectively) and their abundance is largely (though not entirely) determined *in utero* (for a review see [11]), we expect the composition of the muscle to be different in L and N piglets. As such, we believe a lower percentage of type II (fast contracting) fibers in L piglets to be the cause of the observed force deficit.

By investigating both force generating capacity and muscle composition, this study may help to explain the earlier observed differences in locomotion between L and N piglets, more specifically the apparent force deficit in L piglets.

As such, this paper addresses the following questions:

- Do L piglets have shorter legs than N piglets? In other words, are L piglets only more slender (lower BM and BMI) or are they overall smaller (lower BM, BMI and shorter limb lengths)? To answer this question the BM, BMI and the skeletal hind limb length (SHLL) were measured. We hypothesize that L piglets have a lower BM and BMI and a shorter SHLL than N piglets (both at birth and during early development), indicating they are not only more slender (as indicated by the BM and BMI), but are also overall smaller (i.e. have shorter legs, indicated by the SHLL).
- Is there a difference in absolute force generating capacity between L and N piglets? We hypothesize a smaller PCSA for L piglets (because of their smaller size) leading to a reduced absolute force generating capacity both at birth and during early development. To this end, we calculated the maximal isometric force generating capacity (F_iso-max_) of the hind limb.
- Is there a difference in normalized (or relative) force generating capacity between L and N piglets? We hypothesize a smaller PCSA relative to body weight (BW = BM x g; g = 9.81 ms^-2^), indicating a developmental retardation of L piglets’ muscles, both at birth and during early development. Accordingly, the maximal isometric force generating capacity of the hind limb is normalized to BW, thus yielding a dimensionless variable indicating normalized force generating capacity (F’_iso-max_) of the hind limb.
- Is there a difference in muscle composition between L and N piglets? We hypothesize a lower percentage of fast contracting (type II) fibers in the muscles of L piglets. To this end, we calculated the ratio of type II muscle fiber to total muscle fiber (F_type II_/ F_total_), the ratio of type II muscle fiber to total muscle tissue (F_type II_/ T_total_) and the ratio of total other tissue to total muscle tissue (T_other_/T_total_) in m. vastus lateralis.

## Material and methods

### Selection

Institutional and national guidelines for the care and use of animals were followed and all experimental procedures involving animals were approved by the Ethical Committee of Animal Experimentation, University of Antwerp, Belgium (approval number 2015–26).

Thirty-two piglets (Topigs x German Piètrain) were selected from 10 litters in a local farm in October 2016. The mean number of piglets born per litter was 18.2 (± 4.2) (mean ± SD, here and throughout). Between 2 and 6 healthy piglets were selected per litter, in sex-matched (both piglets being male and both piglets being female) pairs of L and N piglets (for an overview of the selected piglets see Table 1). Selected piglets were ear-notched upon selection and remained with the sow for the entire studied period. Because of large between-litter variation in BM at birth and to be able to refer to our study on spatio-temporal gait variables, the same selection procedure was followed as in [13]. All piglets from the abovementioned 10 litters were weighed immediately after birth to calculate the mean BM at birth per litter. In addition, a vitality score was given to each piglet, based on respiration (0–2, no to regular respiration) and locomotion (0–2, no movement to taking a few steps). Piglets scoring 1 or 2 (out of 4) were considered to be low in vitality, while piglets that scored 3 or 4 were considered to have a normal vitality.

**Table 1:** Selected piglets, including category (N or L piglet), age (0, 4, 8 and 96 h) and sex.

Age	N piglets		L piglets		Total
	Male	Female	Male	Female	
**0**	2	3	1	3	9
**4**	2	2	2	2	8
**8**	2	2	2	2	8
**96**	2	2	1	2	7
**Total**	8	9	6	9	32

Combining BM and vitality at birth allowed us to classify piglets into L (n = 15) and N piglets (n = 17). The latter piglets had both a normal vitality and a BM at birth within the limits of the mean BM at birth of the litter at birth ± 1 SD. L piglets, on the other hand, had a BM at birth that was lower than the mean BM of the litter– 1 SD, combined with a low vitality score. The mean BM at birth of the L piglets was 0.79 kg (± 0.26), compared to 1.37 kg (± 0.29) for N piglets.

This study focused on 4 time points in early development: 0, 4, 8 and 96 h after birth. In our earlier studies [13, 23], we found 0, 4 and 8 h after birth to be important in the locomotor development of the young piglet. Within 4 h after birth all spatio-temporal gait variables seemed to reach stable (i.e. mature) values, while the variability of the gait pattern (indicated by left-right symmetry) led to a stable gait pattern within 8 h after birth [13, 23]. Consistent with these former studies, 96 h after birth was chosen as a reference age (control). Though still being within the time frame of early development, at this age piglets seem to show an adult gait pattern with minimal variation. In addition, this is a particularly relevant age in early development, with mortality rates being highest during the first 3 days of life [7, 24]. For each developmental stage we aimed at including 8 piglets with an equal distribution of L piglets/N piglets and females/males. However, one male L piglet that was assigned to the 96 h group died before reaching this age. In addition, our selection procedure posed some challenges with regard to the 0 h group. For one euthanized N piglet at 0 h, there was no L piglet littermate within the litter. Thus another couple was selected, leading to 5 N piglets and 4 L piglets of which 3 males and 6 females, being included in the 0 h group.

### Sampling

The selected piglets were deeply anesthetized with a combination of Zoletil 100 (Tiletamine 50 mg/ml, Zolazepam 50 mg/ml) and Sedaxyl (Xylazine hydrochloride 20 mg/ml), in a dosage of 0.22 ml/kg BM (administered intramuscularly). Euthanasia of the anesthetized animals took place by transecting the jugular veins and carotid arteries.

Immediately after euthanasia, the hind quarter was dissected behind the floating ribs. An important reason to choose the hind limb is that the pelvic anatomy of cursorial quadrupeds appears to be specialized to provide force and to achieve a high power output, creating the (main) horizontal (acceleratory) impulses necessary for forward movement (e.g. [25–27]). In addition, most studies regarding muscle composition (see Introduction) studied the hind limb, which facilitates comparison.

All right legs were frozen at -18°C awaiting dissection. The left hind limb was used for taking tissue samples. For fiber typing, a tissue sample was taken from the proximal part of the m. vastus lateralis (the lateral part of m. quadriceps femoris) of the left hind limb and fixated for 24 h in 4% paraformaldehyde solution (in 0.01 M phosphate-buffered saline solution (PBS), pH = 7.4) at room temperature (± 21°C). After fixation, tissue samples were rinsed with PBS and further processed for paraffin embedding. The m. vastus lateralis is a parallel-fibered muscle that originates laterally, proximally on the femur, converges with the other muscular parts of the m. quadriceps femoris and inserts indirectly via the patella tendon onto the proximal tibia. This muscle was chosen because it is a major extensor of the knee. In addition, its lateral position allowed for a quick and accurate sampling.

### Muscle dissection

We chose to focus on the most important extensors of the hind limb, because they generate the necessary force for support against gravity as well as for propulsion [27]: the hamstrings (m. semitendinosus, m. semimembranosus and m. biceps femoris), mm. glutei (consisting of the m. gluteus superficialis, medius, accessorius and profundus), m. quadriceps femoris (consisting of m. rectus femoris, m. vastus lateralis, m. vastus intermedius and m. vastus medialis), and m. gastrocnemius. The hamstrings and mm. glutei are the main extensors of the hip, while the m. quadriceps femoris is the main extensor of the knee and the m. gastrocnemius of the tarsal joint. After careful consideration, the extensors of the digits (m. extensor digitorum longus, m. extensor digitorum brevis and m. extensor digitorum lateralis) were not included in the force calculations. Their size did not allow for an accurate macroscopic measurement (cf. [28]) of the fiber length, so including them would have introduced a larger error. Additionally, because of their small size, their contribution to the total force generating capacity of the hind limb would have been minimal, compared to the other extensors.

Before dissection, right hind limbs (in a sealed plastic bag) were defrosted in water of ± 38°C. After removal, the abovementioned muscles were temporarily stored in PBS. Each muscle (or muscle bundle, if relevant) and the remaining hind limb skeletal structure were weighted individually (Sartorius BP 210 S, d = 0.1 mg).

### Fiber length

**Fig 1:**
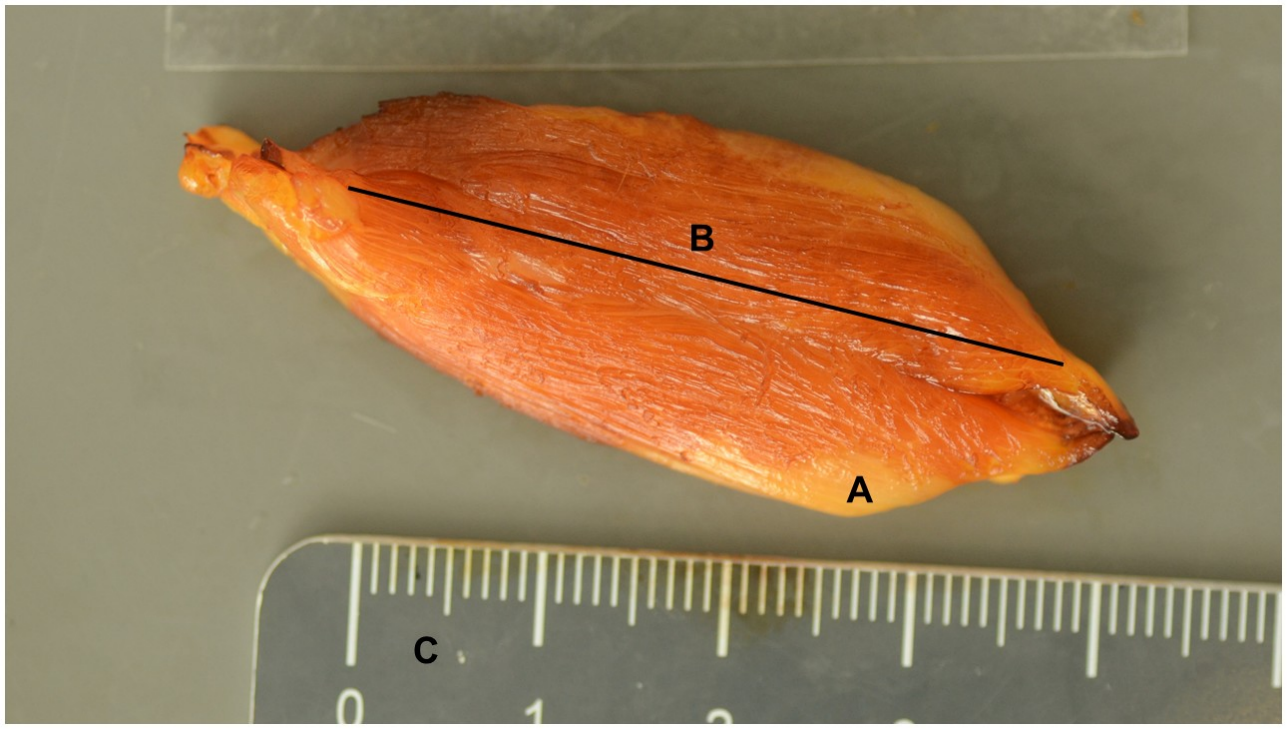
Fascicle length of a parallel-fibered muscle. A. M. semitendinosus. B. Fascicle length. C. Scale (in cm). Category = L piglet, Age = 96 h, Sex = female.

**Fig 2:**
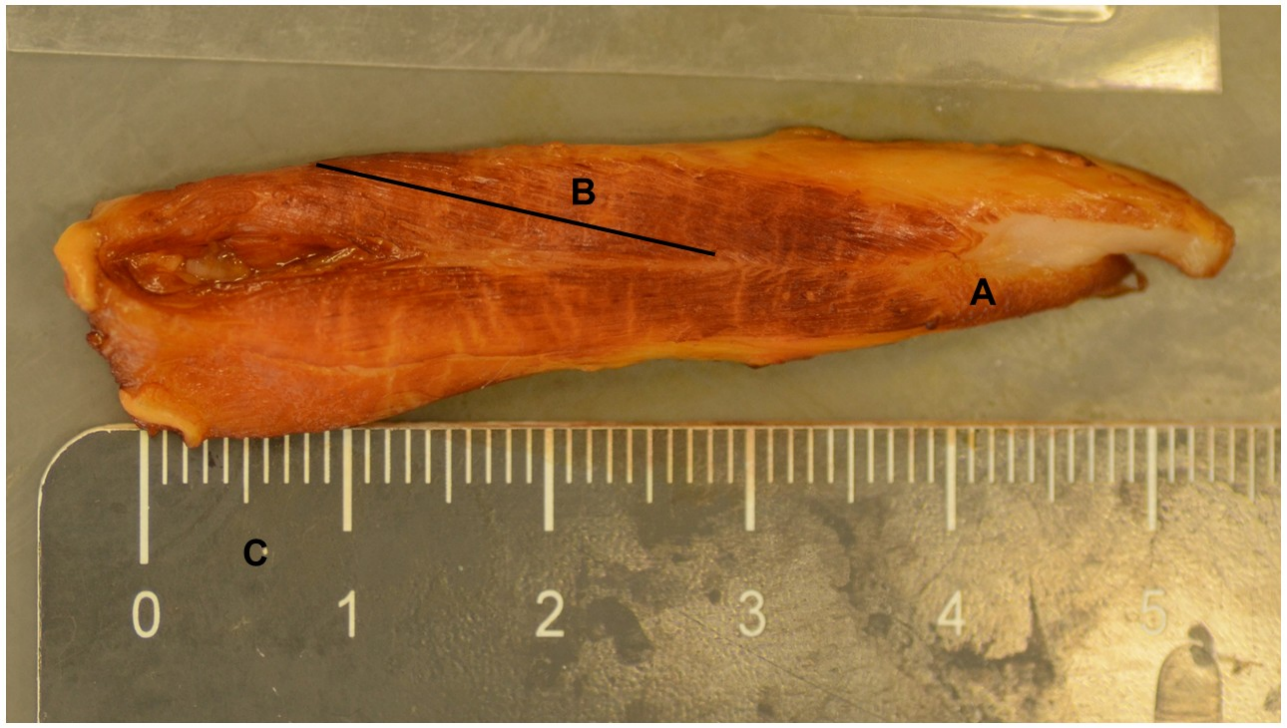
Fascicle length of a pennate-fibered muscle. A. M. gluteus superficialis. B. Fascicle length. C. Scale (in cm). Category = L piglet, Age = 96 h, Sex = female.

The abovementioned muscles (or muscle bundles) were cut along the line of the tendon to reveal the orientation of the fascicles [29]. To enhance visualization of the individual fibers, sodium hypochlorite (NaClO) was applied to the surface of the muscle, hence removing the connective tissue between muscle fibers [30]. To improve the contrast between the fascicles and the connective tissue, the muscles were stained with Lugol’s solution (iodine 1.0 gm, potassium iodide 2.0 gm, distilled water 100 ml) [30, 31]. Afterwards, muscles were photographed with a Nikon D7000 (AF Tamron 90 mm 1:2.8 macro lens, diameter of 55 mm, Tokyo, Japan). From these photographs, the fascicle length was determined with ImageJ (Rasband, W.S., ImageJ, U.S. National Institutes of Health, Bethesda, M.D. USA) (Figs 1 and 2). Five different fascicle lengths were measured per muscle (bundle) at randomly distributed positions. From these 5 measurements, the mean fiber length per muscle (bundle) was calculated.

**Fig 3:**
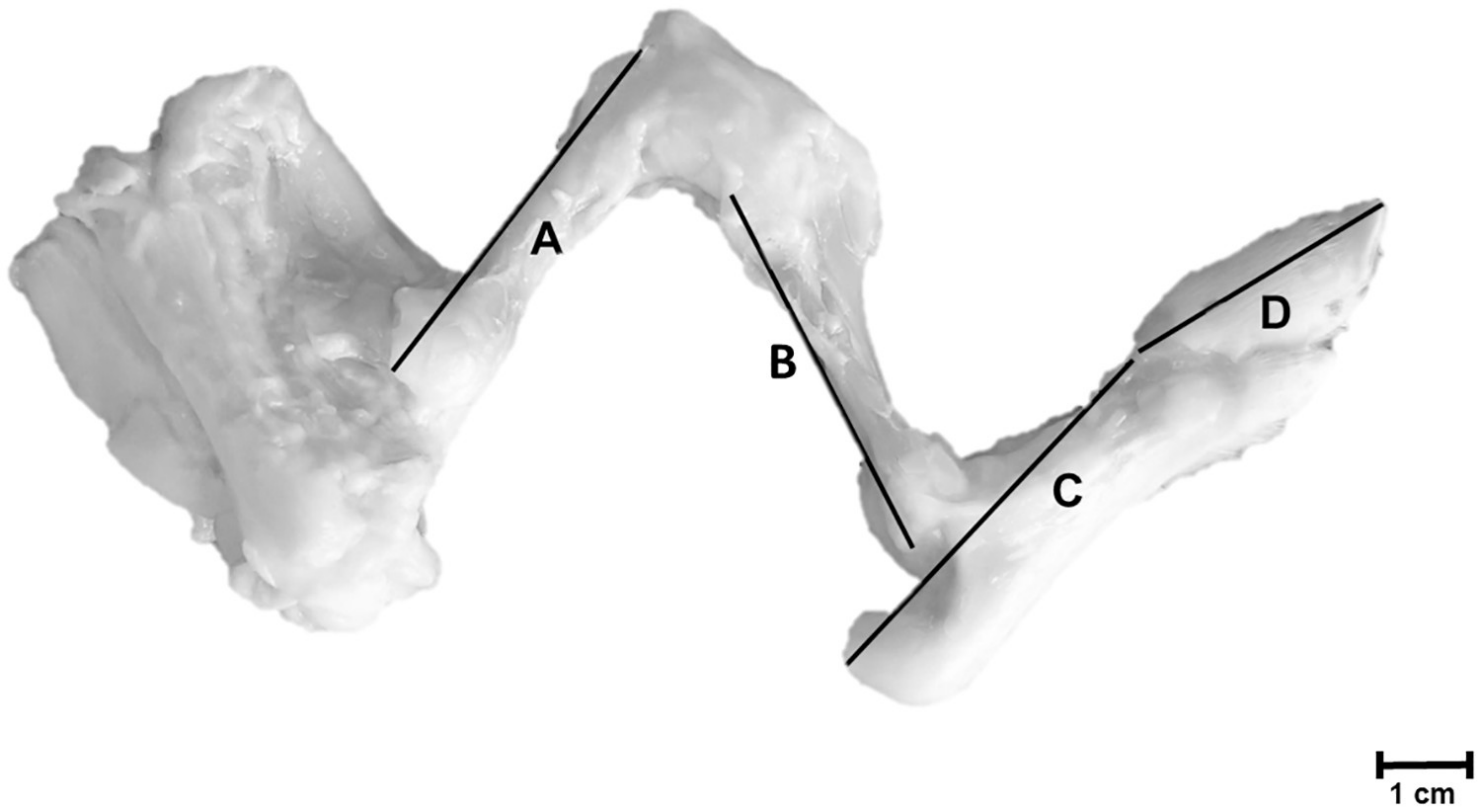
Skeletal hind limb length (SHLL). A. Femur. B. Tibia/fibula C. Tarsals/metatarsals D. Phalanges. E. Scale. Category = L piglet, Age = 96 h, Sex = female.

In addition, the appendicular skeleton of the hind limb (after complete dissection) was photographed (C-8080 Wide Zoom Olympus camera, Olympus Corporation, Tokyo, Japan). The length of the femur, tibia, tarsals/metatarsals and phalanges were measured from these photographs using ImageJ (Fig 3) and summed to get the SHLL. Easily distinguishable landmarks were chosen to measure the lengths of the individual bones (Table 2). We preferred to use distances between specific anatomical landmarks representative for the overall length of the bones, rather than the proximal and distal most ends of the bones (more configuration dependent).

**Table 2:** Landmarks used for determination of bone lengths.

Bone	Proximal landmark	Distal landmark
**Femur**	Most proximal end of trochanter major	Intersection of condylus lateralis and proximal end of patella
**Tibia/fibula**	Most proximal end of condylus lateralis	Most distal point of the malleolus lateralis
**Tarsals/metatarsals**	Tuber calcanei	Most distal end of os metatarsale IV
**Phalanges**	Most proximal end of proximal phalanx	Most distal end of distal phalanx

From the BM and the SHLL, the BMI was calculated. The formula for BMI was adapted from Baxter et al. [32], including the SHLL instead of crown-rump length.

$$BMI=BM.{SHLL}^{-2}$$

### Force calculations

F_iso-max_ was estimated for each muscle by multiplying the PCSA with the maximum isometric stress of a muscle (σ = 0.3 MPa) [27, 33, 34].

$$F_{iso-max}=\sigma .\;PCSA$$

The PCSA of a muscle is obtained by dividing muscle volume (V) by mean fiber length (l). Muscle volume is calculated by dividing muscle mass (m) by mammalian skeletal muscle density (ρ = 1.06 x 10^3^ kg m^-3^, see Méndez and Keys [35]). We assumed muscle density to be the same for L and N piglets, since these groups show no difference in the amount of intramuscular fat, thus leading to the same muscle to fat ratio within a muscle [36, 37].

$$V=m.\rho^{-1}$$

$$PCSA=V.l^{-1}$$

F_iso-max_ was normalized according to body weight (BW = BM x g; g = 9.81 ms^-2^) at time of euthanasia leading to F’_iso-max_.

$${F\prime }_{iso-max}=F_{iso-max}.\;{\left(BM.g\right)}^{-1}$$

F_iso-max_ and F’_iso-max_ were calculated per muscle and summed to get an approximation of the total F_iso-max_ and F’_iso-max_ of the hind limb.

### Immunohistochemistry

The muscle tissue was immunohistochemically stained for type II muscle fibers with a rabbit polyclonal anti-MYH1 antibody (Proteintech, Rosemont, IL, USA).

Ten 4 μm cross-sections per muscle sample were made and mounted onto a microscopic slide using STA-ON 1% adhesive (Leica Biosystems, Wetzlar, Germany). The slides were put in xylol to dissolve the remaining paraffin and the tissue was rehydrated, using a graded alcohol series. Antigen retrieval was performed by incubating the slides for 5 min in hot (below boiling point) sodium citrate buffer (10 mM sodium citrate, 0.05% Tween 20, pH = 6). Cross-sections were rinsed in distilled water and tris buffered saline (TBS, pH = 7.4) consecutively and then exposed to 3% hydrogen peroxide (H_2_O_2_ (Thermo Fisher Scientific, Geel, Belgium), in TBS) for 30 min at room temperature. To block non-specific binding, 10% normal goat serum (in TBS containing 0.3% Triton X (TX; Sigma-Aldrich, St. Louis, MO, USA) and 1% bovine serum albumin (BSA, Sigma-Aldrich, St. Louis, MO, USA)) was applied to the slides for 30 min at room temperature. The primary antibody was applied overnight (in TBS containing 0.3% TX and 1% BSA) at 4°C. After washing in TBS (2 times for 5 min), sections were incubated for 1 h with a biotinylated secondary antibody (a biotinylated goat anti-rabbit antibody, Dako/Agilent, Santa Clara, CA, USA) in a 1/200 dilution (in TBS containing 0.3 TX and 1% BSA) at room temperature. After another washing in TBS (2 times for 5 min), the slides were exposed to a Streptavidine/Horseradish Peroxidase-complex (Dako/Agilent, Santa Clara, CA, USA) in a 1/200 dilution (in TBS containing 0.3 TX and 1% BSA) for 30 min at room temperature. After washing in TBS, the positive reaction was visualized using diaminobenzidine (DAB, Sigma-Aldrich, St. Louis, MO, USA) and the sections were counterstained with hematoxylin (Klinipath, Leuven, Belgium). Afterwards, slides were dehydrated through a graded alcohol series, immersed in xylol and mounted.

### Image analysis

Images were analyzed under a light microscope (Model BX 51, Olympus, Tokyo, Japan) combined with a digital color camera (Model PM-C35, Olympus, Tokyo, Japan) and a motorized stage (Model Cs152DP/A, Prior, UK). With Visiopharm software (Visiopharm, Lund, Sweden) each histological preparation was first scanned in full at a total magnification of 40X, in order to delineate the region of interest. This was done to make sure only m. vastus lateralis was included in the analysis. In each section four random fields were analyzed at a 100X total magnification, leading to a total of 40 analyzed fields per muscle per pig. A preliminary analysis showed that the coefficient of error (CE) of the estimated volume densities was sufficiently low (< 0.05; [38]) when analyzing this amount of fields of view per muscle per pig.

The volume densities of the type II fibers as well as unstained non-muscle fiber tissue in the samples were estimated using a grid overlay on a series of blinded, immunohistochemically stained sections and subsequently counting the number of grid points (Q) within the phase of interest (Q(Y), see further) and the number of grid points falling on the reference area (Q(ref)). The following equation yields the volume density expressed as a percentage [39]:

$$Vv(Y;ref)=\frac{Q(Y)}{Q(ref)}100\%$$

In this study, the number of grid points falling on type II fibers (F_type II_, stained), all muscle fibers (F_total_, stained + not-stained muscle fibers) as well as unstained, non-muscle fiber tissue (T_other_, such as connective tissue, fat, nerves and capillaries) were counted. Although there was no difference in color between the non-stained muscle fibers and T_other_, it was easy to distinguish them from each other because of their typical histologic structure and morphology. For the purpose of this study we did not further differentiate the other tissue (into connective tissue, fat tissue, nerves and blood vessels). We will refer to the total muscle tissue as T_total_ (F_total_ + T_other_). From these data, three ratio’s were calculated: F_type II_/ F_total_; F_type II_/ T_total_ and T_other_ /T_total_.

### Statistics

Linear mixed models were fitted to evaluate the effect of age (0, 4, 8 and 96 h) and birth weight category (L or N piglet) on each of the outcome variables. Sex was added as a covariate. As such, the starting model included age, birth weight category, sex and the interaction between age and birth weight category as fixed effects. Interactions between age and sex or sex and birth weight category and the interaction between age, birth weight category and sex were not included, because this would have made the starting model too complex for the number of observations. To account for the dependence between littermates, sow was added as a random factor. This starting model was simplified using stepwise backwards modelling, during which all non-significant effects were removed from the starting model. To meet normality and/or homoscedasticity assumptions F’_iso-max_ was log transformed, while all other outcome variables required no transformations. Effects were considered statistically significant if *p* ≤ 0.05. Models were fitted using JMP Pro 13 (SAS Institute Inc., Cary, NC, USA). *Post-hoc* analysis with Tukey’s correction was used to compare different age groups. All values are indicated as mean ± SD.

## Results

The following sections highlight only the significant results of this study. Calculations per individual muscle for F_iso-max_ and F’_iso-max_ can be found in S1 Table. For more detailed information on means (± SD) across the different groups, we refer to S2 Table.

### Morphometrics

**Fig 4:**
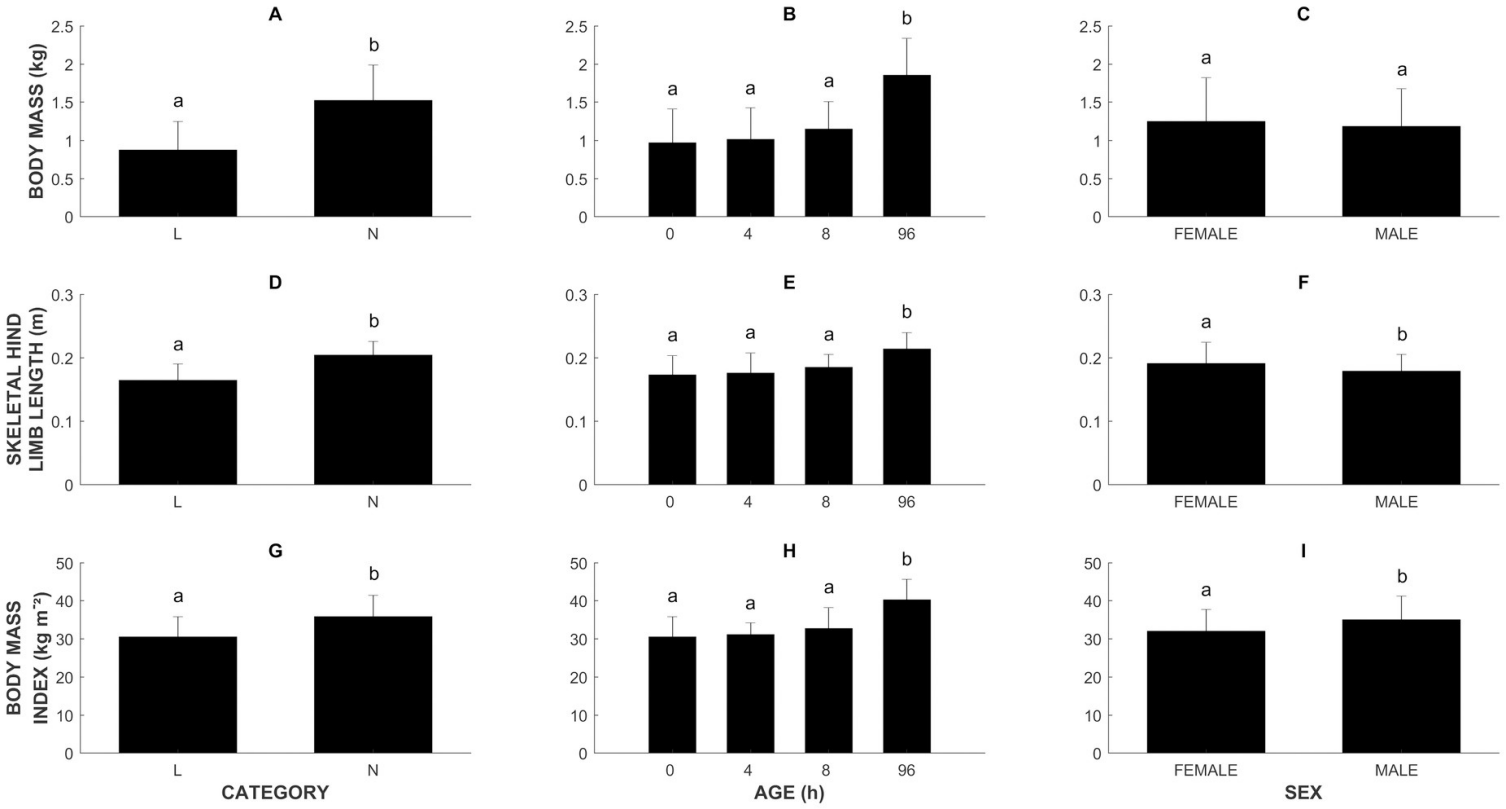
Morphometrics. A-C. Body mass (BM, n = 32). D-F. Skeletal hind limb length (SHLL, n = 32). G-I. Body mass index (BMI, n = 32). All values are mean ± SD. Significant differences (linear mixed models, *p* ≤ 0.05) are indicated by different letters.

Overall, the BM of L piglets was lower than the BM of N piglets (0.87 kg (± 0.37) vs 1.52 kg (± 0.46), *p* < 0.0001; Fig 4A). For both groups, the BM at 96 h was higher than the BM at 0, 4 and 8 h (*p* < 0.0001; Fig 4B). For L piglets, BM increased from 0.65 kg (± 0.28) at 0 h to 1.44 kg (± 0.21) at 96 h. N piglets had a BM of 1.23 kg (± 0.36) at birth that increased to 2.16 kg (± 0.38) by the age of 96 h. BM did not differ significantly between sexes (Fig 4C).

Across all time points, the SHLL was shorter in L piglets (0.16 m (± 0.03)), compared to N piglets (0.20 m (± 0.02) (*p* < 0.0001; Fig 4D). At 96 h, the SHLL was longer than at 0, 4 and 8 h of age (0.21 m (± 0.03) vs 0.17 m (± 0.03), 0.18 m (± 0.03) and 0.19 m (± 0.02), respectively) (*p* < 0.0001 (0 h), *p* = 0.0004 (4 h) and *p* = 0.0006 (8 h); Fig 4E). L piglets showed an increase in SHLL from 0.15 m (± 0.03) to 0.20 m (± 0.02) between 0 and 96 h, respectively. For N piglets this amounted to an increase from 0.19 m (± 0.02) to 0.23 m (± 0.02). The SHLL was longer for females compared to males across all ages (0.19 m (± 0.03) vs 0.18 m (± 0.03), respectively, *p* = 0.0031; Fig 4F).

Similar to the BM and the SHLL, L piglets have a lower BMI than N piglets (30.53 kg m^-2^ (± 5.28) vs 35.80 kg m^-2^(± 5.62), *p* < 0.0001; Fig 4G). The BMI was higher at 96 h (40.19 kg m^-2^ (± 5.44)), compared to 0, 4 and 8 h (30.53 kg m^-2^ (± 5.24), 31.14 kg m^-2^ (± 3.06) and 32.67 kg m^-2^ (± 5.48), respectively) (*p* < 0.0001 (0, 4 h) and *p* = 0.0001 (8 h); Fig 4H). In addition, males had a higher BMI, compared to females (34.99 kg m^-2^ (± 6.22) vs 32.04 kg m^-2^ (± 5.65), *p* = 0.0048); Fig 4I).

### Absolute force generating capacity (F_iso-max_)

**Fig 5:**
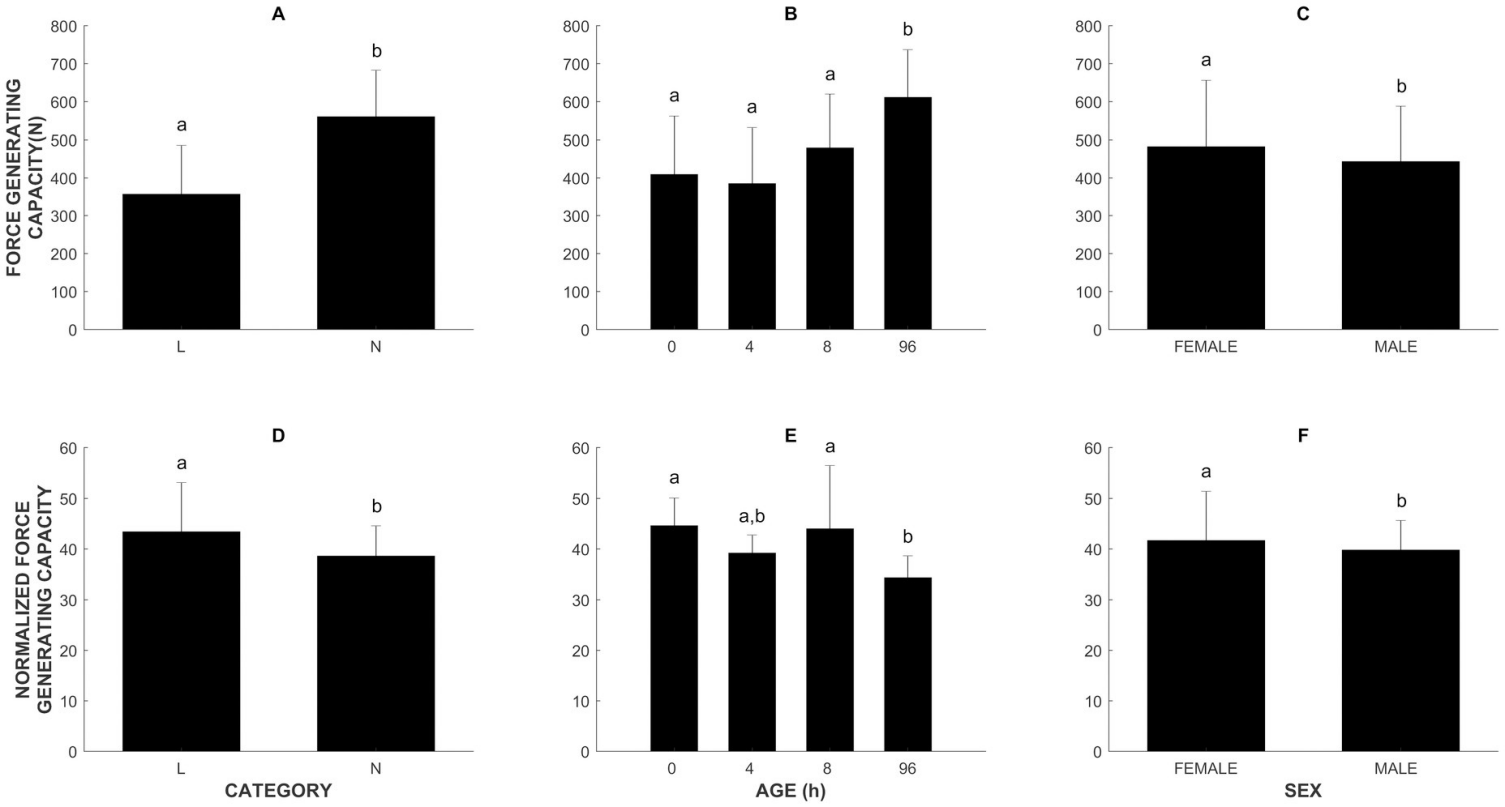
Force generating capacity. A-C. Absolute force generating capacity (F_iso-max_, n = 32). D-F. Normalized force generating capacity (F’_iso-max_, n = 32). All values are mean ± SD. Significant differences (linear mixed models, *p* ≤ 0.05) are indicated by different letters.

L piglets had a lower F_iso-max_ than N piglets (*p* < 0.0001), with L piglets having an F_iso-max_ of 356.37 N (± 128.56), while we noted an F_iso-max_ of 559.59 N (± 122.29) for N piglets (Fig 5A). Piglets that were 96 h old showed an F_iso-max_ that was larger than that of piglets at 0, 4 and 8 h of age (610.59 N (± 125.48) vs 408.42 N (± 152.92), 384.84 N (± 146.20) and 478.73 N (± 140.65), respectively) (*p* < 0.0001 (0, 4 h) and *p* = 0.0038 (8 h); Fig 5B). In L piglets the F_iso-max_ increased from 294.88 N (± 104.32) to 537.05 N (± 42.60) between 0 and 96 h, while for N piglets it increased from 499.25 N (± 124.11) to 665.74 N (± 144.3). In addition, females showed a higher F_iso-max_ (481.23 N (± 174.09)) than males (442.611 N (± 144.84)) (*p* = 0.020; Fig 5C).

### Normalized force generating capacity (F’_iso-max_)

With a value of 43.41 (± 9.62) compared to 38.61 (± 5.94), L piglets had a higher F’_iso-max_ than N piglets (*p* = 0.0107; Fig 5D). At 96 h, F’_iso-max_ was smaller than at 0 and 8 h (34.29 (± 4.31) vs 44.64 (± 5.44) and 44.02 (± 12.39), respectively) (*p* = 0.0026 (0 h) and *p* = 0.0075 (8 h); Fig 5E). In addition, F’_iso-max_ was higher for females than for males (41.69 (± 9.62) vs 39.79 (± 5.84), *p* = 0.0053; Fig 5F).

### Muscle composition

**Fig 6:**
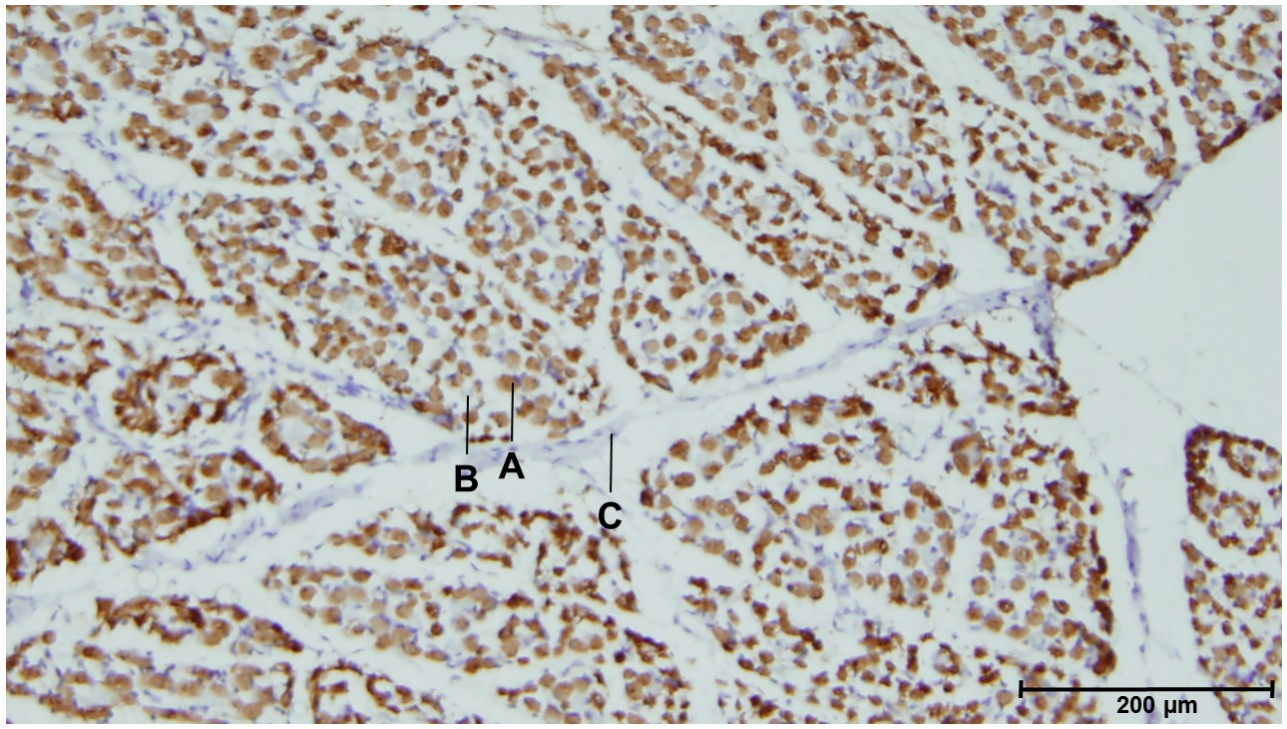
Type II staining of m. quadriceps femoris. A. Type II fiber (stained). B. Type I fiber (not stained). C. Connective tissue. Category = L piglet, Age = 0 h, Sex = female.

The large abundance of type II fibers (stained brown) can be clearly seen in Fig 6. Of the total muscle tissue, 95.58% (± 1.55) consisted of muscle fibers (F_total_/T_total_). Furthermore, F_type II_/ F_total_; F_type II_/ T_total_ and T_other_/T_total_ did not differ significantly with category, age or sex. The mean value for F_type II_/ F_total_ was 90.13% (± 1.69), while F_type II_/ T_total_ was 86.13% (± 2.31). Ratio’s for all piglets separately, including the CE, can be found in S3 Table.

## Discussion

### Do L piglets have shorter legs than N piglets?

Our results show that a lower BM and BMI are associated with a shorter SHLL, thereby confirming our hypothesis. This indicates that L piglets are not only more slender, but also overall smaller (and as such have shorter legs) than N piglets. This shorter SHLL is consistent with our previous data, where stride and step length of L piglets was shorter than that of N piglets [13]. When combining this, we can safely say that shorter legs in L piglets lead to shorter steps.

### Is there a difference in absolute force generating capacity (F_iso-max_) between L and N piglets?

The difference in size between L and N piglets reduces the F_iso-max_ in L piglets, both at birth and during early development, thus confirming our hypothesis. In other words, because of their decreased muscle mass, L piglets have a smaller PCSA and hence a reduced F_iso-max_.

In the light of the two aspects of locomotion, maintaining posture and forward propulsion, these findings make sense. A higher force generating capacity allows N piglets to prevent joint collapse under the increased gravitational load due to a higher BM, thus being able to maintain posture.

However, the fact that a lower BM is associated with a lower muscle mass and hence a reduced absolute force generating capacity does not tell us much about muscle development. To check whether the decreased muscularity of L piglets is still sufficient given their lower BM to achieve a certain level of performance, we have to look at the relative or normalized force generating capacity.

### Is there a difference in normalized force generating capacity (F’_iso-max)_ between L and N piglets?

Unexpectedly, F’_iso-max_ is higher for L than N piglets, thereby rejecting our original hypothesis that L piglets have lesser developed muscles than N piglets. In other words, L piglets have a larger normalized force generating capacity, indicating that the growth of their muscles (given their BW) is not lagging behind. On the contrary, relative to their BM, L piglets have a larger PCSA. This is in accordance with results by Wank et al. [40] indicating that the hind limb plantar flexors of newborn IUGR piglets exhibit a higher specific force (force normalized for muscle mass of the plantar flexor group) than those of N piglets. A study by Bauer et al. [41] also stated that IUGR piglets experience an accelerated muscle development (hemodynamics and contractile function).

Alternatively, this observed difference might have been caused by a non-linear relationship between F_iso-max_ and BM. If an increase in BM is not accompanied by a directly proportional increase in F_iso-max_ (in other words, if F_iso-max_ would increase more slowly, compared to BM), then piglets with a low BM would show a relatively higher FGC, compared to animals with a higher BM. This would explain why L piglets have a higher F’_iso-max_, compared to N piglets and why young piglets (0–8 h) have a higher F’_iso-max_, compared to 96 h piglets. However, a regression showed a linear relationship between BM and F_iso-max_ (R^2^ = 0.85, *p* < 0.0001), so in all likelihood we can dismiss this theory and state that L piglets do in fact have more voluminous muscles (given their BM) than N piglets.

However, this higher F’_iso-max_ for L piglets raises some questions. If L piglets indeed show an accelerated muscle development, it appears as though they do not exploit their full potential. As such, we must look for other factors explaining the observed force deficit in L piglets, such as the composition of the muscles.

### Is there a difference in muscle composition between L and N piglets?

No differences between groups could be discovered, thereby rejecting our hypothesis that L and N piglets have a different muscle composition.

So far, several studies have reported differences between the muscles of L and N piglets with regard to fiber composition. For example, studies by Wank et al. [40] and Bauer et al. [42] revealed an increased proportion of type I fibers in the hind limb plantar flexors and m. gastrocnemius in 1-day-old IUGR piglets compared to N piglets. On the other hand, looking at m. semitendinosus, Rehfeldt and Kuhn [6] observed no differences in the percentages of fiber types between L and N piglets. Similarly, Gondret et al. [43] found the relative proportions of type I and II fibers in m. semitendinosus and m. rhomboideus to be independent of birth weight. As such, at this point, it is hard to say whether there is an unambiguous link between IUC (and the associated differences in BM) and the fiber composition of pig muscles. Given the great structural and functional diversity in muscles, different results might be obtained for other muscles.

In addition, differences might be more subtle than what we investigated. In our study we only distinguished between type I and II fiber. However, in pigs type II fibers actually comprise three subtypes, type IIa, IIb and IIx [44], though not all subtypes are expressed in each muscle [45]. Type IIa is considered to have oxidative glycolytic properties, type IIb is considered glycolytic [46], while the somewhat later discovered type IIx has properties intermediate between those of IIa and IIb [47]. Considering that these subtypes have a different ATPase activity and contraction speed, having a different composition with regard to these three subtypes might have an effect on force production [20, 48].

Furthermore, it might also be possible that the myofibrillar structure of the fibers of L piglets is not as mature as in N piglets. This is not visible in an enzymatic study such as ours, but can be investigated in the future by means of ultrastructural methods.

### Other possible explanations for the observed force deficit in L piglets

We proposed that the observed force deficit in L piglets was attributed to a lesser growth and maturation of the musculoskeletal system and/or different fiber composition of the muscles. However, our results indicate that L piglets have shorter legs, but that their muscles can generate relatively larger forces than those of N piglets. In addition, we see no difference in the fiber composition of m. vastus lateralis. As such, we must look to other factors that might explain the observed force deficit for L piglets.

One option is that L and N piglets differ in the development of the control (neural) system, such as the degree of myelination. It is a known fact that changes in force are mediated by the manner in which the nervous system recruits motor units within a muscle [14].

Alternatively, we might consider that, though the muscles of L piglets might be sufficiently developed, L piglets might lack the energy required to fully exploit the capacity of their muscles. As mentioned in the introduction of this paper, an increase in performance requires an animal’s muscles to generate larger forces and contract more rapidly. However, this requires a increased energy supply [14]. To understand this, one must consider the energy balance of a newborn piglet. Being devoid of brown fat [36, 49–51] piglets are largely dependent on (a limited amount of) glycogen pools in the muscles and the liver for initial energy provision [36, 51, 52] and on a rapid ingestion of colostrum [53]. As such, a different glycogen concentration in the muscles and liver might be an explanation for the observed force difference between L and N piglets. So far, no differences between L and N piglets have been detected with regard to glycogen concentration [36, 52], though our preliminary results (data not shown) do indicate that there is a difference. However, even when accounting for a possible equal glycogen concentration in L and N piglets, it is possible that, because of their size difference, L and N piglets have to allocate their available energy in a different manner. A newborn pig relies mainly on shivering thermogenesis to maintain a stable body temperature. As small animals are more prone to heat loss, due to their larger surface to mass ratio, L piglets might be forced to allocate more of their available energy towards maintaining homeothermy than N piglets, leaving less energy available for locomotion. This theory is supported by findings from Baxter et al. [54], who state that low weight piglets have a lower average rectal temperature during the first day after birth and by the statement by Herpin et al. [55] that a higher birth weight has a positive effect on thermoregulation. After this initial period, energy levels are replenished with fatty acids and lactose from milk [49, 56, 57]. However, due to extensive teat competition in large litters [4, 58], the intake of milk is reduced in L piglets and as such the amount of dietary energy for locomotion in the L piglets is less [59].

### On the effect of sex

Unexpectedly, we came across differences in both morphometrics and force generating capacity (both absolute and relative) between sexes. In spite of not having a different BM, females had a higher SHLL than males. This implies a different body build, with females having longer hind limbs than males. In addition, females exhibit a higher F_iso-max_, indicating they have more muscular hind limbs. Even when normalized to body weight, F’_iso-max_, is higher for females than males, indicating that males have less voluminous muscles, given their BM. From our earlier study, we see that this is not reflected in their gait pattern [23]. It might, however, be reflected in a larger male mortality rate [60]. If males possess smaller muscles than females, this could imply a smaller absolute amount of glycogen (relative to their BM, which is not lower), thereby increasing the need to suckle and thus to spend more time in the vicinity of the sow and are hence more prone to crushing by the sow. This need is even more amplified because males have more trouble maintaining homeothermy and as such need to allocate more of the available energy towards countering hypothermia [60]. In addition, if males have a lesser force generating capacity than females, chances are they will often lose the competition for a functional teat, thereby again, having to spend more time around the udder. Combined with being less able to rapidly move away, their risk to be crushed by the sow further increases.

Alternatively, if these differences in body build persist later during ontogeny and remain independent from locomotor performance, they most likely represent a sexual dimorphism related to the pelvic differences in function of child bearing. As stated by Glucksmann [61] in their review on sexual dimorphisms in mammals, the female pelvic region is often enlarged for the purpose of child bearing and for the accommodation of the genital tract (which in males is partially located on the exterior of the body). This broader pelvis can in turn affect the hip joint and the skeleton. It is not hard to imagine that as a ‘by-product’ of this sexual dimorphism, the muscles in the pelvic area of females might be more developed than those in males.

## Conclusions

Our previous study showed a reduced motor performance in L piglets, indicating that they experience a force deficit. We tried to explain this force deficit by investigating the force generating capacity of the hind limb and the composition of m. quadriceps femoris. Our results show that L piglets have a lower absolute force generating capacity due to a lesser muscle mass. However, L piglets do show a larger normalized force generating capacity, suggesting they actually have more voluminous muscles, given their BM, than N piglets. In addition, no differences in muscle fiber composition of the m. vastus lateralis were detected between L and N piglets, thereby indicating that the observed force deficit cannot be explained by a difference in type II fiber percentage. Ultrastructual, neural and energetic studies will be key to further unravel the effect of IUC on locomotory performance in piglets.

## Supporting information

S1 Table Calculations F_iso-max_ and F’_iso-max_ per individual muscle. (PDF)

S2 Table Means (± SD) by category, sex and age. (PDF)

S3 Table F_type II_/ F_total_; F_type II_/ T_total_, T_other_ /T_total_ and CE per piglet. (PDF)
