# Supplementary material for: How does intrauterine crowding affect locomotor performance in newborn pigs? A study of force generating capacity and muscle composition of the hind limb
Source: PLoS One. 2018 Dec 14;13(12):e0209233. doi: 10.1371/journal.pone.0209233 (PMC6294349; doi:10.1371/journal.pone.0209233)
Supplement: S1 Table — (PDF) [file pone.0209233.s001.pdf]

m. biceps femoris

PIGLET	SOW	CATEGORY	GENDER	AGE (in h)	BM (in kg)	FIBER LENGTH (in m)	MUSCLE MASS (in kg)	PCSA (in m^2)	$F_{iso-max}$ (in N)	$F'_{iso-max}$
1	2264	L	M	0	0.525	0.018832	0.0024662	0.000124013	37.20396134	7.223719498
2	2264	N	M	0	0.94	0.030722	0.007126	0.000219651	65.8951832	7.145897933
3	1954	N	F	0	1.37	0.034188	0.0077083	0.000213511	64.05340922	4.765985046
4	1819	N	F	0	1.62	0.0382754	0.0108843	0.000269288	80.78637145	5.083397607
5	1997	L	F	0	0.795	0.022424	0.0039822	0.000168169	50.45071433	6.468911114
6	1997	N	M	0	1.458	0.037918	0.0089357	0.000223161	66.94844497	4.680734013
7	2264	N	M	4	1.14	0.03414	0.0065123	0.000180637	54.19113144	4.845675862
8	1954	L	F	0	0.955	0.032294	0.005332	0.000156352	46.90570159	5.00671946
9	2105	L	M	4	0.545	0.021432	0.0022219	9.81743E-05	29.45229521	5.508757252
10	2264	L	M	4	0.7	0.022974	0.0040811	0.00016822	50.46589227	7.349045038
11	2105	N	M	4	1.22	0.035728	0.0077817	0.000206254	61.87612593	5.170044445
12	2353	L	F	0	0.315	0.02013	0.0012526	5.89257E-05	17.67770853	5.72066357
13	2353	N	F	0	0.77	0.025234	0.0038821	0.000145686	43.70568749	5.785997259
14	1954	N	M	8	1.35	0.034052	0.0080591	0.00022412	67.23590525	5.076898497
15	1954	L	M	8	0.705	0.026254	0.0032098	0.000115776	34.73280262	5.022057767
16	2264	N	F	8	1.2	0.037404	0.0106812	0.00027042	81.12586403	6.891425759
17	2264	L	F	8	0.65	0.028458	0.0063375	0.000210887	63.26608111	9.921756624
18	1819	L	M	8	1.1	0.034178	0.007651	0.000211986	63.59586709	5.893417393
19	1819	N	M	8	1.648	0.03924	0.0129723	0.000313057	93.91724006	5.80923716
20	2321	L	F	4	0.5	0.021468	0.0026547	0.000117101	35.13024671	7.162129808
21	2321	N	F	4	1.38	0.032138	0.0085294	0.000251325	75.39750451	5.569405997
22	1870	L	F	4	0.975	0.031506	0.0045501	0.000136761	41.02844047	4.289546561
23	1870	N	F	4	1.65	0.044894	0.012749	0.00026892	80.67614826	4.984162621
24	1196	L	F	8	1	0.033828	0.005803	0.000162447	48.73417126	4.967805429
25	1196	N	F	8	1.52	0.032444	0.010048	0.000293279	87.98377064	5.900515762
26	2105	L	M	96	1.2	0.023904	0.0059138	0.000234278	70.28350143	5.970395976
27	1997	L	F	96	1.52	0.032926	0.0106857	0.000307327	92.19796596	6.183135225
28	1997	N	F	96	2.63	0.043928	0.0176942	0.000381439	114.4318285	4.435290618
29	2105	N	M	96	2.265	0.035898	0.0159352	0.000420362	126.1085702	5.675542605
30	2342	L	F	96	1.605	0.03481	0.0105073	0.00028584	85.75203703	5.44628547

31	2342	N	F	96	2.005	0.04108	0.016	0.000368829	110.6488448	5.625530711
32	2353	N	M	96	1.73	0.030248	0.0083627	0.00026181	78.54294649	4.627986453

m. gluteus superficialis

PIGLET	SOW	CATEGORY	GENDER	AGE (in h)	BM (in kg)	FIBER LENGTH (in m)	MUSCLE MASS (in kg)	PCSA (in m^2)	$F_{iso\text{-}max}$ (in N)	$F'_{iso\text{-}max}$
1	2264	L	M	0	0.525	0.011716	0.0004973	4.01953E-05	12.05858732	2.341359609
2	2264	N	M	0	0.94	0.014116	0.0012089	8.10989E-05	24.32966138	2.638391283
3	1954	N	F	0	1.37	0.012736	0.0015505	0.000115286	34.58565912	2.573395174
4	1819	N	F	0	1.62	0.01737	0.001867	0.000101784	30.53527503	1.921400123
5	1997	L	F	0	0.795	0.015194	0.0006775	4.22254E-05	12.66760504	1.624270579
6	1997	N	M	0	1.458	0.019516	0.0017561	8.52108E-05	25.5632325	1.787266185
7	2264	N	M	4	1.14	0.014626	0.0014983	9.70084E-05	29.10251669	2.602295964
8	1954	L	F	0	0.955	0.010376	0.0009028	8.23944E-05	24.7183185	2.638435884
9	2105	L	M	4	0.545	0.01188	0.0003736	2.97801E-05	8.934037343	1.671022331
10	2264	L	M	4	0.7	0.015214	0.0008884	5.5297E-05	16.58908661	2.415769129
11	2105	N	M	4	1.22	0.014872	0.0015881	0.000101122	30.33652318	2.534760714
12	2353	L	F	0	0.315	0.008338	0.0002546	2.89156E-05	8.674687629	2.807206003
13	2353	N	F	0	0.77	0.014526	0.0006672	4.34957E-05	13.04870264	1.727458416
14	1954	N	M	8	1.35	0.018544	0.0013177	6.72898E-05	20.18693868	1.524290307
15	1954	L	M	8	0.705	0.01628	0.0005455	3.17305E-05	9.519139491	1.376383845
16	2264	N	F	8	1.2	0.016946	0.0014449	8.07433E-05	24.2229998	2.057679222
17	2264	L	F	8	0.65	0.015718	0.0008487	5.1132E-05	15.33960775	2.405646945
18	1819	L	M	8	1.1	0.018792	0.0010175	5.1274E-05	15.38221052	1.42546664
19	1819	N	M	8	1.648	0.01887	0.001612	8.08964E-05	24.26892133	1.501150582
20	2321	L	F	4	0.5	0.01649	0.0006086	3.495E-05	10.48500469	2.137615634
21	2321	N	F	4	1.38	0.02284	0.0016676	6.91404E-05	20.74211909	1.532163209
22	1870	L	F	4	0.975	0.013502	0.0012942	9.07694E-05	27.23081429	2.846996972
23	1870	N	F	4	1.65	0.026464	0.0021949	7.85408E-05	23.56224064	1.455672359
24	1196	L	F	8	1	0.01854	0.0008218	4.19752E-05	12.59255173	1.283644417
25	1196	N	F	8	1.52	0.021636	0.0015013	6.57093E-05	19.71277879	1.322011561
26	2105	L	M	96	1.2	0.012814	0.0010907	8.0604E-05	24.18120451	2.054128823
27	1997	L	F	96	1.52	0.016746	0.0015596	8.81938E-05	26.45815011	1.77438101
28	1997	N	F	96	2.63	0.017854	0.0026198	0.000138953	41.68597309	1.615716604

29	2105	N	M	96	2.265	0.019194	0.0023654	0.000116701	35.01034888	1.575648081
30	2342	L	F	96	1.605	0.016954	0.0018052	0.00010083	30.24896243	1.921172841
31	2342	N	F	96	2.005	0.021522	0.0021103	9.28534E-05	27.85600992	1.416235655
32	2353	N	M	96	1.73	0.015696	0.0014327	8.64375E-05	25.93125927	1.527947727

m. gluteus medius

PIGLET	SOW	CATEGORY	GENDER	AGE (in h)	BM (in kg)	FIBER LENGTH (in m)	MUSCLE MASS (in kg)	PCSA (in m^2)	$F_{iso-max}$ (in N)	$F'_{iso-max}$
1	2264	L	M	0	0.525	0.013718	0.0013914	9.605E-05	28.81499423	5.594872916
2	2264	N	M	0	0.94	0.02093	0.0033418	0.000151198	45.35953177	4.918942001
3	1954	N	F	0	1.37	0.021824	0.0050015	0.000217021	65.10633623	4.844329578
4	1819	N	F	0	1.62	0.020464	0.0059704	0.00027628	82.88391144	5.215383108
5	1997	L	F	0	0.795	0.016254	0.0018952	0.000110416	33.12471336	4.247329879
6	1997	N	M	0	1.458	0.02372	0.0043648	0.000174255	52.27655987	3.654941828
7	2264	N	M	4	1.14	0.021268	0.0040288	0.000179385	53.81537778	4.812076629
8	1954	L	F	0	0.955	0.0194	0.0028527	0.000139248	41.77454311	4.45901907
9	2105	L	M	4	0.545	0.014946	0.0010685	6.76995E-05	20.30985791	3.798755794
10	2264	L	M	4	0.7	0.013192	0.0012692	9.11078E-05	27.3323364	3.980244124
11	2105	N	M	4	1.22	0.021314	0.0033324	0.000148057	44.4170285	3.711253864
12	2353	L	F	0	0.315	0.011588	0.0006703	5.47768E-05	16.43304594	5.317879695
13	2353	N	F	0	0.77	0.019296	0.0019871	9.75188E-05	29.25565119	3.87302265
14	1954	N	M	8	1.35	0.028158	0.0036969	0.000124329	37.29866048	2.816374862
15	1954	L	M	8	0.705	0.017094	0.0014593	8.0842E-05	24.25259528	3.506711964
16	2264	N	F	8	1.2	0.020334	0.0051864	0.000241535	72.46036643	6.155314851
17	2264	L	F	8	0.65	0.018768	0.0026916	0.000135809	40.74270518	6.389509164
18	1819	L	M	8	1.1	0.02005	0.0034338	0.00016218	48.65393335	4.508751121
19	1819	N	M	8	1.648	0.022934	0.0057362	0.000236854	71.05617305	4.395169201
20	2321	L	F	4	0.5	0.012118	0.0008433	6.59003E-05	19.77008282	4.030597925
21	2321	N	F	4	1.38	0.023206	0.0045908	0.000187337	56.20117838	4.151426257
22	1870	L	F	4	0.975	0.015444	0.002131	0.000130665	39.19954204	4.0983342
23	1870	N	F	4	1.65	0.02106	0.0058955	0.000265093	79.52791807	4.913225099
24	1196	L	F	8	1	0.01816	0.002361	0.000123116	36.93494694	3.765030269
25	1196	N	F	8	1.52	0.019724	0.0042999	0.000206443	61.93279761	4.153441548
26	2105	L	M	96	1.2	0.019386	0.002882	0.00014078	42.23408645	3.587672991

27	1997	L	F	96	1.52	0.02424	0.005102	0.000199317	59.795042	4.010075782
28	1997	N	F	96	2.63	0.026374	0.0095015	0.000341155	102.3466206	3.966877154
29	2105	N	M	96	2.265	0.027556	0.0080473	0.000276548	82.96431894	3.733826543
30	2342	L	F	96	1.605	0.021804	0.0051307	0.000222831	66.84944172	4.245743375
31	2342	N	F	96	2.005	0.028106	0.0071697	0.000241567	72.47016975	3.684477377
32	2353	N	M	96	1.73	0.02053	0.0038572	0.000177918	53.37532657	3.145034651

m. gluteus accessorius

PIGLET	SOW	CATEGORY	GENDER	AGE (in h)	BM (in kg)	FIBER LENGTH (in m)	MUSCLE MASS (in kg)	PCSA (in m^2)	$F_{iso-max}$ (in N)	$F'_{iso-max}$
1	2264	L	M	0	0.525	0.007188	0.0003648	4.80599E-05	14.41796934	2.799469801
2	2264	N	M	0	0.94	0.007512	0.0011548	0.000145575	43.67254817	4.735999758
3	1954	N	F	0	1.37	0.011564	0.0017956	0.000147041	44.11221345	3.282232003
4	1819	N	F	0	1.62	0.012752	0.0021193	0.00015738	47.21407337	2.970895998
5	1997	L	F	0	0.795	0.005348	0.0007545	0.000133599	40.07976644	5.1391234
6	1997	N	M	0	1.458	0.008896	0.0014992	0.000159588	47.87647155	3.347307453
7	2264	N	M	4	1.14	0.012598	0.0011285	8.48274E-05	25.44821328	2.275534567
8	1954	L	F	0	0.955	0.011182	0.0010566	8.94803E-05	26.84407571	2.865339429
9	2105	L	M	4	0.545	0.0091	0.0003597	3.74313E-05	11.2293956	2.100346137
10	2264	L	M	4	0.7	0.00836	0.0006868	7.77965E-05	23.33895172	3.398711478
11	2105	N	M	4	1.22	0.012408	0.0014417	0.00011003	33.00885426	2.758046679
12	2353	L	F	0	0.315	0.00711	0.0003054	4.06757E-05	12.20272344	3.948909741
13	2353	N	F	0	0.77	0.008634	0.0007681	8.42445E-05	25.27336429	3.345825793
14	1954	N	M	8	1.35	0.010516	0.0011615	0.000104594	31.37805163	2.369317146
15	1954	L	M	8	0.705	0.011056	0.0005566	4.7674E-05	14.30218886	2.067970714
16	2264	N	F	8	1.2	0.009452	0.0018376	0.000184104	55.2312161	4.691744487
17	2264	L	F	8	0.65	0.010164	0.001024	9.54051E-05	28.62151622	4.488593464
18	1819	L	M	8	1.1	0.01557	0.0013953	8.48624E-05	25.45870555	2.359253596
19	1819	N	M	8	1.648	0.010912	0.0017417	0.000151149	45.34467892	2.804788488
20	2321	L	F	4	0.5	0.009018	0.0003366	3.5346E-05	10.60379242	2.161833316
21	2321	N	F	4	1.38	0.016684	0.0013075	7.42126E-05	22.26377749	1.644563924
22	1870	L	F	4	0.975	0.01164	0.0007231	5.88276E-05	17.6482935	1.845139026
23	1870	N	F	4	1.65	0.014368	0.0018871	0.000124375	37.31263603	2.305170113
24	1196	L	F	8	1	0.010296	0.0006867	6.31589E-05	18.94767165	1.931465

25	1196	N	F	8	1.52	0.007548	0.0012856	0.000161291	48.38729103	3.24502998
26	2105	L	M	96	1.2	0.010292	0.0011642	0.000107118	32.13550684	2.729825589
27	1997	L	F	96	1.52	0.01205	0.001402	0.000110179	33.05356469	2.216693807
28	1997	N	F	96	2.63	0.013212	0.0031035	0.000222443	66.73298035	2.58651955
29	2105	N	M	96	2.265	0.01815	0.0028022	0.000146204	43.86113198	1.973979427
30	2342	L	F	96	1.605	0.013124	0.0020359	0.000146902	44.07045732	2.799003961
31	2342	N	F	96	2.005	0.017524	0.0020402	0.000110249	33.07477019	1.681564193
32	2353	N	M	96	1.73	0.010308	0.0016206	0.00014888	44.6641179	2.631744056

m. gluteus profundus

PIGLET	SOW	CATEGORY	GENDER	AGE (in h)	BM (in kg)	FIBER LENGTH (in m)	MUSCLE MASS (in kg)	PCSA (in m^2)	$F_{iso-max}$ (in N)	$F'_{iso-max}$
1	2264	L	M	0	0.525	0.006506	0.000341	4.96337E-05	14.89010144	2.891141487
2	2264	N	M	0	0.94	0.007914	0.0007318	8.75654E-05	26.26961426	2.848766376
3	1954	N	F	0	1.37	0.008766	0.0009718	0.000104981	31.49435837	2.343382543
4	1819	N	F	0	1.62	0.010086	0.0015996	0.000150186	45.05570277	2.835082794
5	1997	L	F	0	0.795	0.007536	0.0005287	6.64362E-05	19.93084709	2.555580826
6	1997	N	M	0	1.458	0.00823	0.0009779	0.00011252	33.75607533	2.360072889
7	2264	N	M	4	1.14	0.009742	0.0010068	9.78658E-05	29.35975439	2.625297708
8	1954	L	F	0	0.955	0.007608	0.0006264	7.79682E-05	23.39045024	2.496699088
9	2105	L	M	4	0.545	0.00668	0.0002577	3.6532E-05	10.95961486	2.049886347
10	2264	L	M	4	0.7	0.007678	0.000465	5.7351E-05	17.20529731	2.505504195
11	2105	N	M	4	1.22	0.00686	0.0009415	0.000129967	38.99002783	3.257802162
12	2353	L	F	0	0.315	0.00397	0.0001189	2.83614E-05	8.508415388	2.753398828
13	2353	N	F	0	0.77	0.009176	0.0006279	6.47997E-05	19.43991737	2.573562277
14	1954	N	M	8	1.35	0.007488	0.0008518	0.000107723	32.31685849	2.44020527
15	1954	L	M	8	0.705	0.007622	0.0003576	4.44288E-05	13.32864197	1.927204397
16	2264	N	F	8	1.2	0.00867	0.0010695	0.000116815	35.04443221	2.976931041
17	2264	L	F	8	0.65	0.008548	0.0005846	6.47635E-05	19.42905305	3.04697766
18	1819	L	M	8	1.1	0.0088156	0.0009776	0.000105014	31.50406923	2.919476344
19	1819	N	M	8	1.648	0.009072	0.0014744	0.000153903	46.17103575	2.85590267
20	2321	L	F	4	0.5	0.00617	0.0002003	3.0742E-05	9.222594666	1.880243561
21	2321	N	F	4	1.38	0.009794	0.0010689	0.000103351	31.00518406	2.290267552
22	1870	L	F	4	0.975	0.006404	0.0005125	7.57842E-05	22.73525779	2.376984008

23	1870	N	F	4	1.65	0.010398	0.0012974	0.000118157	35.44715767	2.189921087
24	1196	L	F	8	1	0.008134	0.0006328	7.36713E-05	22.10139258	2.252945217
25	1196	N	F	8	1.52	0.009206	0.0005403	5.55776E-05	16.67329113	1.118172322
26	2105	L	M	96	1.2	0.008196	0.000898	0.000103755	31.12660278	2.644121881
27	1997	L	F	96	1.52	0.012742	0.0008782	6.52667E-05	19.58002169	1.313108381
28	1997	N	F	96	2.63	0.014748	0.0019517	0.000125319	37.59562159	1.457177692
29	2105	N	M	96	2.265	0.011184	0.0015142	0.00012821	38.46302348	1.731036424
30	2342	L	F	96	1.605	0.0126256	0.0013812	0.000103595	31.07863101	1.973866771
31	2342	N	F	96	2.005	0.017708	0.0017344	9.27504E-05	27.8251227	1.414665309
32	2353	N	M	96	1.73	0.011178	0.0010252	8.68522E-05	26.05564502	1.53527691

m. semitendinosus

PIGLET	SOW	CATEGORY	GENDER	AGE (in h)	BM (in kg)	FIBER LENGTH (in m)	MUSCLE MASS (in kg)	PCSA (in m^2)	$F_{iso-max}$ (in N)	$F'_{iso-max}$
1	2264	L	M	0	0.525	0.01961	0.01961	4.32293E-05	12.9688007	2.518091492
2	2264	N	M	0	0.94	0.03167	0.03167	7.55722E-05	22.67165657	2.458591599
3	1954	N	F	0	1.37	0.035224	0.035224	7.35794E-05	22.07382492	1.642434349
4	1819	N	F	0	1.62	0.034538	0.034538	0.000105656	31.69680694	1.994488299
5	1997	L	F	0	0.795	0.02824	0.02824	5.48431E-05	16.45292783	2.10963371
6	1997	N	M	0	1.458	0.034382	0.034382	8.47982E-05	25.4394477	1.778611709
7	2264	N	M	4	1.14	0.02594	0.02594	8.89874E-05	26.69622205	2.387129321
8	1954	L	F	0	0.955	0.028498	0.028498	5.95437E-05	17.86309566	1.906708686
9	2105	L	M	4	0.545	0.02366	0.02366	3.24075E-05	9.722248905	1.818449421
10	2264	L	M	4	0.7	0.028788	0.028788	4.88518E-05	14.6555304	2.134196942
11	2105	N	M	4	1.22	0.033552	0.033552	7.693E-05	23.07899949	1.928360112
12	2353	L	F	0	0.315	0.01998	0.01998	2.45037E-05	7.351101101	2.37888164
13	2353	N	F	0	0.77	0.02259	0.02259	5.61726E-05	16.85178478	2.23093117
14	1954	N	M	8	1.35	0.027866	0.027866	8.11412E-05	24.34234779	1.838060014
15	1954	L	M	8	0.705	0.024862	0.024862	3.90336E-05	11.71009427	1.693176635
16	2264	N	F	8	1.2	0.032968	0.032968	9.31028E-05	27.93083789	2.372650178
17	2264	L	F	8	0.65	0.0288	0.0288	6.39764E-05	19.19290562	3.00994364
18	1819	L	M	8	1.1	0.027782	0.027782	0.000100642	30.1924562	2.797929404
19	1819	N	M	8	1.648	0.039922	0.039922	9.57952E-05	28.73856862	1.777619962
20	2321	L	F	4	0.5	0.021046	0.021046	3.78995E-05	11.36984571	2.318011357

21	2321	N	F	4	1.38	0.031724	0.031724	8.94374E-05	26.83120608	1.981947295
22	1870	L	F	4	0.975	0.028924	0.028924	5.77172E-05	17.31516608	1.810310366
23	1870	N	F	4	1.65	0.03913	0.03913	9.12895E-05	27.38685733	1.691956713
24	1196	L	F	8	1	0.030864	0.030864	6.3475E-05	19.04248551	1.941130021
25	1196	N	F	8	1.52	0.035054	0.035054	9.87248E-05	29.61745385	1.986255556
26	2105	L	M	96	1.2	0.028198	0.028198	8.05586E-05	24.16757475	2.052971012
27	1997	L	F	96	1.52	0.034874	0.034874	0.000101236	30.37069815	2.036770894
28	1997	N	F	96	2.63	0.041804	0.041804	0.000147672	44.30170884	1.717100531
29	2105	N	M	96	2.265	0.037382	0.037382	0.00014145	42.43504409	1.909798043
30	2342	L	F	96	1.605	0.040174	0.040174	9.71154E-05	29.134628	1.850399205
31	2342	N	F	96	2.005	0.046456	0.046456	9.71186E-05	29.1355848	1.481290901
32	2353	N	M	96	1.73	0.030376	0.030376	8.8038E-05	26.41140135	1.556239142

m. semimembranosus

PIGLET	SOW	CATEGORY	GENDER	AGE (in h)	BM (in kg)	FIBER LENGTH (in m)	MUSCLE MASS (in kg)	PCSA (in m^2)	$F_{iso-max}$ (in N)	$F'_{iso-max}$
1	2264	L	M	0	0.525	0.02774	0.0014669	5.00761E-05	15.0228174	2.916910324
2	2264	N	M	0	0.94	0.036292	0.004141	0.000108051	32.41542088	3.515238562
3	1954	N	F	0	1.37	0.03714	0.0055389	0.000141227	42.36809737	3.152458565
4	1819	N	F	0	1.62	0.048478	0.0074581	0.000145687	43.70597816	2.750152789
5	1997	L	F	0	0.795	0.026932	0.002661	9.35648E-05	28.06943075	3.599129467
6	1997	N	M	0	1.458	0.049664	0.0061466	0.0001172	35.16013978	2.458238757
7	2264	N	M	4	1.14	0.04144	0.0047889	0.000109434	32.83018713	2.935617713
8	1954	L	F	0	0.955	0.040862	0.0041883	9.70631E-05	29.1189358	3.108158231
9	2105	L	M	4	0.545	0.026602	0.0015532	5.52903E-05	16.58709871	3.102450919
10	2264	L	M	4	0.7	0.030692	0.0028301	8.73198E-05	26.19593646	3.81475702
11	2105	N	M	4	1.22	0.043836	0.0047454	0.000102513	30.75383247	2.569628889
12	2353	L	F	0	0.315	0.020132	0.0008935	4.20285E-05	12.60854497	4.080237195
13	2353	N	F	0	0.77	0.03182	0.0027518	8.18941E-05	24.56823896	3.252477456
14	1954	N	M	8	1.35	0.042088	0.0053267	0.000119849	35.95483381	2.714904203
15	1954	L	M	8	0.705	0.032844	0.0016757	4.83144E-05	14.49431057	2.095749824
16	2264	N	F	8	1.2	0.04277	0.0064024	0.000141755	42.52662231	3.612523132
17	2264	L	F	8	0.65	0.030072	0.0030824	9.7065E-05	29.11950712	4.566691308
18	1819	L	M	8	1.1	0.035414	0.0046707	0.000124894	37.46832917	3.472183224

19	1819	N	M	8	1.648	0.045906	0.0073507	0.000151634	45.49006765	2.813781487
20	2321	L	F	4	0.5	0.028478	0.0016549	5.50299E-05	16.50895588	3.36574024
21	2321	N	F	4	1.38	0.04734	0.0061472	0.000122966	36.88981065	2.724948711
22	1870	L	F	4	0.975	0.03793	0.0032135	8.0229E-05	24.06870911	2.516397094
23	1870	N	F	4	1.65	0.050288	0.0068859	0.000129668	38.90036571	2.403259859
24	1196	L	F	8	1	0.03955	0.0038088	9.11964E-05	27.35892426	2.788881168
25	1196	N	F	8	1.52	0.040004	0.0060815	0.000143961	43.18815278	2.896356616
26	2105	L	M	96	1.2	0.037764	0.0041572	0.000104246	31.27377204	2.656623517
27	1997	L	F	96	1.52	0.042424	0.0067501	0.000150673	45.2018208	3.031400611
28	1997	N	F	96	2.63	0.052116	0.0136309	0.000247679	74.30376032	2.879957222
29	2105	N	M	96	2.265	0.05325	0.0103385	0.000183854	55.15631669	2.48232158
30	2342	L	F	96	1.605	0.045298	0.0074974	0.000156736	47.02068925	2.986379164
31	2342	N	F	96	2.005	0.061266	0.009269	0.000143268	42.98042367	2.185180457
32	2353	N	M	96	1.73	0.034724	0.0061719	0.000168316	50.4947783	2.97530409

m. rectus femoris

PIGLET	SOW	CATEGORY	GENDER	AGE (in h)	BM (in kg)	FIBER LENGTH (in m)	MUSCLE MASS (in kg)	PCSA (in m^2)	$F_{iso\text{-}max}$ (in N)	$F'_{iso\text{-}max}$
1	2264	L	M	0	0.525	0.012634	0.0011277	8.45257E-05	25.35771079	4.92358833
2	2264	N	M	0	0.94	0.022636	0.002999	0.000125462	37.63865685	4.081664047
3	1954	N	F	0	1.37	0.019636	0.0039488	0.000190436	57.13068761	4.250890095
4	1819	N	F	0	1.62	0.023148	0.0049405	0.000202113	60.63379715	3.815318027
5	1997	L	F	0	0.795	0.012438	0.0018996	0.000144626	43.38793141	5.563304216
6	1997	N	M	0	1.458	0.021626	0.0035776	0.000156658	46.99730123	3.28583982
7	2264	N	M	4	1.14	0.022448	0.0030725	0.000129614	38.8840573	3.476944158
8	1954	L	F	0	0.955	0.017974	0.0025235	0.000132952	39.88557968	4.257390917
9	2105	L	M	4	0.545	0.012654	0.0009906	7.41321E-05	22.23964395	4.159702971
10	2264	L	M	4	0.7	0.013144	0.0016485	0.000118767	35.63023917	5.188617907
11	2105	N	M	4	1.22	0.019844	0.0030907	0.00014749	44.24711614	3.697056879
12	2353	L	F	0	0.315	0.010346	0.0007962	7.28762E-05	21.86286312	7.075016785
13	2353	N	F	0	0.77	0.016258	0.0018349	0.000106876	32.06288652	4.244659772
14	1954	N	M	8	1.35	0.018856	0.002751	0.000138158	41.44750164	3.129648631
15	1954	L	M	8	0.705	0.01518	0.001284	8.00994E-05	24.02982393	3.474501187
16	2264	N	F	8	1.2	0.019626	0.004352	0.000209987	62.99621096	5.351360089

17	2264	L	F	8	0.65	0.016022	0.0021589	0.0001276	38.28010633	6.003310018
18	1819	L	M	8	1.1	0.019402	0.0031665	0.00015455	46.36500689	4.296636724
19	1819	N	M	8	1.648	0.01838	0.0045498	0.000234414	70.32409239	4.349886459
20	2321	L	F	4	0.5	0.012982	0.0009489	6.92173E-05	20.76520287	4.23347663
21	2321	N	F	4	1.38	0.022616	0.0037032	0.000155059	46.51775091	3.43613814
22	1870	L	F	4	0.975	0.017516	0.0025826	0.000139623	41.88702796	4.379312367
23	1870	N	F	4	1.65	0.028676	0.0049762	0.000164329	49.29882765	3.045675573
24	1196	L	F	8	1	0.02187	0.0021914	9.48875E-05	28.46624683	2.901758087
25	1196	N	F	8	1.52	0.01698	0.0037351	0.000208305	62.49163454	4.190919211
26	2105	L	M	96	1.2	0.019708	0.0028969	0.000139196	41.7588266	3.547300934
27	1997	L	F	96	1.52	0.017378	0.0036975	0.000201486	60.44574383	4.053714244
28	1997	N	F	96	2.63	0.024714	0.0078598	0.000301165	90.34950746	3.501878174
29	2105	N	M	96	2.265	0.020334	0.0067546	0.000314567	94.37004301	4.247143542
30	2342	L	F	96	1.605	0.023658	0.0047618	0.000190603	57.18083062	3.631670311
31	2342	N	F	96	2.005	0.026882	0.0056797	0.000200078	60.02347803	3.051671434
32	2353	N	M	96	1.73	0.024298	0.0041709	0.000162553	48.76593846	2.873435651

m. vastus lateralis

PIGLET	SOW	CATEGORY	GENDER	AGE (in h)	BM (in kg)	FIBER LENGTH (in m)	MUSCLE MASS (in kg)	PCSA (in m^2)	$F_{iso-max}$ (in N)	$F'_{iso-max}$
1	2264	L	M	0	0.525	0.010236	0.0009161	8.47518E-05	25.42552577	4.936755648
2	2264	N	M	0	0.94	0.02076	0.0021579	9.84328E-05	29.52985418	3.202317889
3	1954	N	F	0	1.37	0.014044	0.0028391	0.000191437	57.43110937	4.273243404
4	1819	N	F	0	1.62	0.023436	0.0035576	0.000143751	43.12518425	2.713606943
5	1997	L	F	0	0.795	0.014694	0.0013336	8.59452E-05	25.78356039	3.306029708
6	1997	N	M	0	1.458	0.02073	0.0030637	0.000139953	41.98597772	2.935470631
7	2264	N	M	4	1.14	0.022886	0.0020971	8.67731E-05	26.03194291	2.327730646
8	1954	L	F	0	0.955	0.019348	0.0016785	8.21526E-05	24.64578204	2.630693335
9	2105	L	M	4	0.545	0.015348	0.0008004	4.93846E-05	14.81537423	2.771067573
10	2264	L	M	4	0.7	0.017066	0.0015296	8.48755E-05	25.46264236	3.707971802
11	2105	N	M	4	1.22	0.01967	0.002545	0.000122524	36.75705967	3.071227057
12	2353	L	F	0	0.315	0.008006	0.000423	5.00335E-05	15.01004928	4.857385331
13	2353	N	F	0	0.77	0.01038	0.0011702	0.000106758	32.02728149	4.239946183
14	1954	N	M	8	1.35	0.010836	0.0021874	0.000191159	57.34777174	4.330257994

15	1954	L	M	8	0.705	0.009118	0.000993	0.00010313	30.93905163	4.473514741
16	2264	N	F	8	1.2	0.020028	0.0029449	0.000139242	41.77248443	3.548461131
17	2264	L	F	8	0.65	0.010762	0.0018219	0.000160313	48.09377693	7.542347201
18	1819	L	M	8	1.1	0.013866	0.00243	0.000165955	49.78659376	4.613714555
19	1819	N	M	8	1.648	0.021372	0.0035178	0.00015587	46.76094891	2.892391662
20	2321	L	F	4	0.5	0.015592	0.0009286	5.63979E-05	16.91937007	3.449412859
21	2321	N	F	4	1.38	0.02162	0.0029252	0.000128126	38.43768396	2.839285849
22	1870	L	F	4	0.975	0.019616	0.0015775	7.61544E-05	22.84631979	2.388595603
23	1870	N	F	4	1.65	0.01781	0.0041287	0.000219526	65.85772804	4.068682423
24	1196	L	F	8	1	0.019542	0.0017121	8.29652E-05	24.88957351	2.537163456
25	1196	N	F	8	1.52	0.020772	0.0030797	0.0001404	42.11991011	2.824716328
26	2105	L	M	96	1.2	0.019682	0.0022862	0.000109997	32.99911779	2.803187036
27	1997	L	F	96	1.52	0.025158	0.0036503	0.000137401	41.22017034	2.764376465
28	1997	N	F	96	2.63	0.03021	0.0067931	0.000212938	63.88142849	2.475995569
29	2105	N	M	96	2.265	0.025954	0.0050619	0.000184691	55.40725024	2.493614897
30	2342	L	F	96	1.605	0.028268	0.002944	9.86231E-05	29.58694058	1.879126493
31	2342	N	F	96	2.005	0.02881	0.0046344	0.00015233	45.69909438	2.3234012
32	2353	N	M	96	1.73	0.025176	0.0031121	0.000117058	35.11754521	2.069231303

m. vastus medius + m. vastus intermedius

PIGLET	SOW	CATEGORY	GENDER	AGE (in h)	BM (in kg)	FIBER LENGTH (in m)	MUSCLE MASS (in kg)	PCSA (in m^2)	$F_{iso-max}$ (in N)	$F'_{iso-max}$
1	2264	L	M	0	0.525	0.0085	0.0010032	0.000111765	33.52941176	6.51024936
2	2264	N	M	0	0.94	0.018816	0.0022248	0.00011197	33.59085111	3.642706217
3	1954	N	F	0	1.37	0.021544	0.0034945	0.000153601	46.08037884	3.428676149
4	1819	N	F	0	1.62	0.020704	0.0044677	0.000204346	61.30375553	3.857474455
5	1997	L	F	0	0.795	0.011486	0.0014904	0.000122877	36.86305859	4.726669434
6	1997	N	M	0	1.458	0.022468	0.003024	0.000127454	38.23619855	2.673302945
7	2264	N	M	4	1.14	0.0181284	0.0022218	0.00011606	34.81792005	3.113357302
8	1954	L	F	0	0.955	0.013548	0.0017243	0.000120524	36.15721542	3.859424929
9	2105	L	M	4	0.545	0.013624	0.0007914	5.50082E-05	16.50246223	3.086620511
10	2264	L	M	4	0.7	0.01396	0.0012313	8.35246E-05	25.05738799	3.64895704
11	2105	N	M	4	1.22	0.015126	0.0026595	0.000166499	49.94974036	4.17353824
12	2353	L	F	0	0.315	0.01001	0.0005402	5.11042E-05	15.33125965	4.961331861

13	2353	N	F	0	0.77	0.013316	0.0014124	0.000100443	30.1329228	3.98916065
14	1954	N	M	8	1.35	0.017768	0.0024486	0.000130501	39.15043899	2.956200324
15	1954	L	M	8	0.705	0.012906	0.0012131	8.90105E-05	26.70313667	3.861038695
16	2264	N	F	8	1.2	0.018084	0.0026805	0.000140365	42.10936086	3.577077885
17	2264	L	F	8	0.65	0.010752	0.0016946	0.00014925	44.77496787	7.021872167
18	1819	L	M	8	1.1	0.016738	0.0023231	0.000131432	39.42953704	3.653927999
19	1819	N	M	8	1.648	0.018362	0.0036784	0.000189703	56.91101187	3.520222323
20	2321	L	F	4	0.5	0.010698	0.0006213	5.49965E-05	16.49894203	3.363698681
21	2321	N	F	4	1.38	0.016702	0.002886	0.00016363	49.0891129	3.626077568
22	1870	L	F	4	0.975	0.016156	0.0016617	9.73991E-05	29.21972417	3.05493862
23	1870	N	F	4	1.65	0.025008	0.0037835	0.000143269	42.9805644	2.655334038
24	1196	L	F	8	1	0.01309	0.0017064	0.000123446	37.03382179	3.775109255
25	1196	N	F	8	1.52	0.019592	0.0031735	0.00015339	46.01686913	3.086060755
26	2105	L	M	96	1.2	0.0147	0.0022168	0.000142806	42.84168213	3.639286623
27	1997	L	F	96	1.52	0.021284	0.0030031	0.000133614	40.0842609	2.688198194
28	1997	N	F	96	2.63	0.024858	0.0062516	0.000238156	71.4467265	2.769220765
29	2105	N	M	96	2.265	0.026364	0.004567	0.000164042	49.21268327	2.214827114
30	2342	L	F	96	1.605	0.027616	0.0039327	0.000134855	40.45641361	2.569468729
31	2342	N	F	96	2.005	0.027198	0.0041681	0.000145123	43.53699971	2.213477505
32	2353	N	M	96	1.73	0.023094	0.0037153	0.000152346	45.7037739	2.693003712

m. gastrocnemius

PIGLET	SOW	CATEGORY	GENDER	AGE (in h)	BM (in kg)	FIBER LENGTH (in m)	MUSCLE MASS (in kg)	PCSA (in m^2)	$F_{iso\text{-}max}$ (in N)	$F'_{iso\text{-}max}$
1	2264	L	M	0	0.525	0.00627	0.0009717	0.000146758	44.02729448	8.548574239
2	2264	N	M	0	0.94	0.009114	0.0023108	0.000240098	72.02954496	7.811128999
3	1954	N	F	0	1.37	0.01052	0.0040377	0.000363458	109.0374395	8.113085821
4	1819	N	F	0	1.62	0.010784	0.0046662	0.000409751	122.9251669	7.734937071
5	1997	L	F	0	0.795	0.00613	0.0015466	0.000238921	71.67618271	9.190491375
6	1997	N	M	0	1.458	0.011682	0.0039558	0.000320666	96.19986459	6.725861645
7	2264	N	M	4	1.14	0.009144	0.0029806	0.000308676	92.60294878	8.280393152
8	1954	L	F	0	0.955	0.010002	0.0022999	0.00021775	65.32500318	6.972797624
9	2105	L	M	4	0.545	0.005662	0.0010108	0.000169056	50.71690055	9.486089003
10	2264	L	M	4	0.7	0.006932	0.0013179	0.000180036	54.0108784	7.865280094

11	2105	N	M	4	1.22	0.010366	0.0034534	0.00031548	94.64398471	7.907954806
12	2353	L	F	0	0.315	0.005564	0.0004903	8.3447E-05	25.03410725	8.101259566
13	2353	N	F	0	0.77	0.00864	0.0019076	0.000209079	62.72359007	8.303690915
14	1954	N	M	8	1.35	0.012802	0.0024486	0.000181124	54.33721294	4.102934491
15	1954	L	M	8	0.705	0.006908	0.0012042	0.000165075	49.52262199	7.160535565
16	2264	N	F	8	1.2	0.0083	0.0034913	0.000398332	119.4995893	10.15117136
17	2264	L	F	8	0.65	0.005284	0.0019915	0.000356906	107.0717346	16.79161524
18	1819	L	M	8	1.1	0.012278	0.0032974	0.00025432	76.29592471	7.070329415
19	1819	N	M	8	1.648	0.01268	0.004885	0.000364822	109.4466949	6.769809318
20	2321	L	F	4	0.5	0.006316	0.0006011	9.0124E-05	27.03721429	5.512174167
21	2321	N	F	4	1.38	0.011368	0.0038713	0.000322484	96.74534979	7.146312531
22	1870	L	F	4	0.975	0.013528	0.0018896	0.000132273	39.68200634	4.148776115
23	1870	N	F	4	1.65	0.013166	0.0056212	0.000404307	121.2921022	7.493411315
24	1196	L	F	8	1	0.008454	0.0018632	0.000208705	62.61156634	6.382422664
25	1196	N	F	8	1.52	0.006678	0.0041629	0.000590317	177.0952449	11.87665948
26	2105	L	M	96	1.2	0.007754	0.0031645	0.00038647	115.9408927	9.848869578
27	1997	L	F	96	1.52	0.009562	0.0040928	0.000405329	121.5987526	8.154860282
28	1997	N	F	96	2.63	0.018366	0.0082377	0.000424744	127.4232648	4.938828805
29	2105	N	M	96	2.265	0.01658	0.0062669	0.000357935	107.3805379	4.832683588
30	2342	L	F	96	1.605	0.015726	0.0050275	0.00030274	90.82201739	5.768290186
31	2342	N	F	96	2.005	0.017686	0.0056999	0.000305192	91.55771643	4.654912994
32	2353	N	M	96	1.73	0.01614	0.0044435	0.00026071	78.21300834	4.608545505
