# Supplementary material for: How does intrauterine crowding affect locomotor performance in newborn pigs? A study of force generating capacity and muscle composition of the hind limb
Source: PLoS One. 2018 Dec 14;13(12):e0209233. doi: 10.1371/journal.pone.0209233 (PMC6294349; doi:10.1371/journal.pone.0209233)
Supplement: S2 Table — (PDF) [file pone.0209233.s002.pdf]

GROUP MEANS (± SD) by CATEGORY

CATEGORY	BM (in kg)	SHLL (in m)	$F_{iso\text{-}max}$ (in N)	$F'_{iso\text{-}max}$
**L**	0.87 ± 0.37	0.16 ± 0.03	356.37 ± 128.55	43.41 ± 9.62
**N**	1.52 ± 0.46	0.20 ± 0.02	559.59 ± 122.29	38.61 ± 5.94

GROUP MEANS (± SD) by GENDER

GENDER	BM (in kg)	SHLL (in m)	$F_{iso\text{-}max}$ (in N)	$F'_{iso\text{-}max}$
**F**	1.25 ± 0.57	0.19 ± 0.03	481.23 ± 174.09	41.69 ± 9.62
**M**	1.18 ± 0.49	0.17 ± 0.03	442.61 ± 144.84	39.79 ± 5.84

GROUP MEANS (± SD) by AGE

AGE (in h)	BM (in kg)	SHLL (in m)	$F_{iso\text{-}max}$ (in N)	$F'_{iso\text{-}max}$
**0**	0.97 ± 0.44	0.17 ± 0.03	408.42 ± 152.92	44.64 ± 5.44
**4**	1.01 ± 0.41	0.18 ± 0.03	384.85 ± 146.20	39.19 ± 3.55
**8**	1.15 ± 0.36	0.19 ± 0.02	478.73 ± 140.65	44.02 ± 12.39
**96**	1.85 ± 0.48	0.21 ± 0.03	610.59 ± 125.48	34.29 ± 4.311

GROUP MEANS (± SD) by AGE, split by CATEGORY

	BM (in kg)		SHLL (in m)		$F_{iso\text{-}max}$ (in N)		$F'_{iso\text{-}max}$	
	CATEGORY		CATEGORY		CATEGORY		CATEGORY	
AGE (in h)	L	N	L	N	L	N	L	N
**0**	0.64 ± 0.28	1.23 ± 0.36	0.15 ± 0.03	0.19 ± 0.02	294.88 ± 104.32	499.25 ± 124.11	47.98 ± 5.40	41.96 ± 4.15
**4**	0.68 ± 0.21	1.35 ± 0.23	0.15 ± 0.02	0.20 ± 0.02	261.15 ± 67.69	508.55 ± 66.99	39.73 ± 5.01	38.65 ± 1.88
**8**	0.86 ± 0.22	1.43 ± 0.20	0.17 ± 0.01	0.20 ± 0.01	377.58 ± 100.36	579.89 ± 93.82	46.35 ± 16.95	41.69 ± 7.53
**96**	1.44 ± 0.21	2.16 ± 0.38	0.20 ± 0.02	0.23 ± 0.02	537.05 ± 42.60	665.74 ± 144.28	38.28 ± 3.23	31.30 ± 1.54

GROUP MEANS (± SD) by AGE, split by GENDER

	BM (in kg)		SHLL (in m)		$F_{iso-max}$ (in N)		$F'_{iso-max}$	
	GENDER		GENDER		GENDER		GENDER	
AGE (in h)	F	M	F	M	F	M	F	M
0	0.97 ± 0.47	0.97 ± 0.47	0.17 ± 0.03	0.17 ± 0.03	411.37 ± 176.10	402.52 ± 126.23	44.64 ± 4.65	44.63 ± 8.03
4	1.13 ± 0.50	0.90 ± 0.33	0.19 ± 0.04	0.17 ± 0.03	404.88 ± 181.74	364.81 ± 125.60	36.88 ± 2.41	41.51 ± 3.05
8	1.09 ± 0.36	1.20 ± 0.40	0.18 ± 0.01	0.19 ± 0.03	508.20 ± 138.03	449.27 ± 157.46	49.93 ± 15.76	38.11 ± 4.13
96	1.94 ± 0.51	1.73 ± 0.53	0.23 ± 0.02	0.20 ± 0.02	635.38 ± 133.41	577.23 ± 132.92	33.84 ± 3.65	34.88 ± 5.91

GROUP MEANS (± SD) by AGE, split by CATEGORY and GENDER

	BM (in kg)				SHLL (in m)			
	CATEGORY				CATEGORY			
	L		N		L		N	
	GENDER		GENDER		GENDER		GENDER	
AGE (in h)	F	M	F	M	F	M	F	M
0	0.69 ± 0.33	0.53 (no SD)	1.25 ± 0.44	1.20 ± 0.37	0.15 ± 0.03	0.14 (no SD)	0.20 ± 0.02	0.18 ± 0.01
4	0.74 ± 0.34	0.63 ± 0.11	1.51 ± 0.19	1.18 ± 0.06	0.16 ± 0.03	0.15 ± 0.01	0.21 ± 0.02	0.19 ± 0.003
8	0.83 ± 0.25	0.90 ± 0.28	1.36 ± 0.27	1.50 ± 0.21	0.17 ± 0.003	0.17 ± 0.02	0.20 ± 0.002	0.21 ± 0.02
96	1.56 ± 0.06	1.2 (no SD)	2.32 ± 0.44	2.00 ± 0.38	0.21 ± 0.02	0.17 (no SD)	0.25 ± 0.007	0.21 ± 0.003

	$F_{iso-max}$ (in N)				$F'_{iso-max}$			
	CATEGORY				CATEGORY			
	L		N		L		N	
	GENDER		GENDER		GENDER		GENDER	
AGE (in h)	F	M	F	M	F	M	F	M
0	305.27 ± 125.21	263.72 (no SD)	517.48 ± 167.56	471.92 ± 54.47	46.91 ± 6.06	51.20 (no SD)	42.38 ± 1.36	41.34 ± 8.00
4	258.59 ± 90.90	263.71 ± 0.71	551.18 ± 72.21	465.92 ± 31.31	36.69 ± 4.14	42.78 ± 4.57	37.07 ± 0.19	40.23 ± 0.78
8	396.32 ± 81.47	358.84 ± 148.92	620.07 ± 21.42	539.71 ± 139.61	52.86 ± 25.92	39.83 ± 4.49	46.99 ± 6.21	36.39 ± 4.38
96	561.10 ± 12.59	488.94 (no SD)	709.65 ± 176.56	621.82 ± 153.51	36.65 ± 2.23	41.53 (no SD)	31.04 ± 1.85	31.56 ± 1.86
