# Supplementary material for: How does intrauterine crowding affect locomotor performance in newborn pigs? A study of force generating capacity and muscle composition of the hind limb
Source: PLoS One. 2018 Dec 14;13(12):e0209233. doi: 10.1371/journal.pone.0209233 (PMC6294349; doi:10.1371/journal.pone.0209233)
Supplement: S3 Table — (PDF) [file pone.0209233.s003.pdf]

PIGLET	CATEGORY	AGE (in h)	GENDER	SOW	$F_{type\ II}/F_{total}$ (in %)	$F_{type\ II}/T_{total}$ (in %)	T_{other}/T_{total} (in %)	CE
1	L	0	M	2264	86.469863	83.73471611	3.116455805	0.012548666
2	N	0	M	2264	90.14309934	85.11005227	5.421287135	0.009449897
3	N	0	F	1954	88.78936829	84.91797709	4.424771079	0.009213509
4	N	0	F	1819	91.32608584	86.6670748	5.103351754	0.009668445
5	L	0	F	1997	92.56544177	88.36373376	4.539414716	0.008471621
6	N	0	M	1997	92.52119143	88.13246975	4.756454506	0.006292039
7	N	4	M	2264	93.14384586	89.36659439	4.06477567	0.008033804
8	L	0	F	1954	87.69178532	84.42966099	3.650757322	0.013728081
9	L	4	M	2105	92.04297377	88.72144824	3.66228921	0.010687386
10	L	4	M	2264	91.14332616	87.73563055	3.858291669	0.012002393
11	N	4	M	2105	89.37357475	87.57974856	1.956807031	0.01290464
12	L	0	F	2353	89.4802648	83.11511724	7.428393374	0.012176443
13	N	0	F	2353	93.75670935	91.25409828	2.657548603	0.009158284
14	N	8	M	1954	89.42417152	84.70882952	5.217387115	0.011585407
15	L	8	M	1954	91.43537479	87.72430713	4.034932037	0.009314339
16	N	8	F	2264	90.83981284	85.19315745	6.225039964	0.011487337
17	L	8	F	2264	89.6031916	83.46804647	6.597438746	0.010065109
18	L	8	M	1819	90.71820097	86.73534073	4.357470174	0.010991486
19	N	8	M	1819	89.82838391	86.32433998	3.733114209	0.010510615
20	L	4	F	2321	88.54503291	84.58082082	4.393688295	0.012655367
21	N	4	F	2321	91.62465683	88.42858912	3.442142073	0.009627505
22	L	4	F	1870	89.98220505	88.22673128	1.980338789	0.00818833
23	N	4	F	1870	91.37508113	88.96596081	2.618153624	0.010517879
24	L	8	F	1196	88.15861239	79.91505198	9.262508673	0.016067062
25	N	8	F	1196	90.15905835	87.11749717	3.346056182	0.011360013
26	L	96	M	2105	90.49909184	84.91746294	6.081131424	0.009176031
27	L	96	F	1997	89.05198862	84.83848052	4.634002706	0.01225857
28	N	96	F	1997	90.11418383	87.56858688	2.843147393	0.012346497
29	N	96	M	2105	88.25307163	84.44413088	4.331356451	0.010088769
30	L	96	F	2342	88.1508507	84.85308659	3.752507329	0.012901833
31	N	96	F	2342	90.01786817	84.84721938	5.665090111	0.01069374
32	N	96	M	2353	88.00709618	84.31710573	4.229864709	0.014757412
